# On the evolutionary conservation of hydrogen bonds made by buried polar amino acids: the hidden joists, braces and trusses of protein architecture

**DOI:** 10.1186/1471-2148-10-161

**Published:** 2010-05-31

**Authors:** Catherine L Worth, Tom L Blundell

**Affiliations:** 1 Biocomputing Group, Biochemistry Department, University of Cambridge, Cambridge, CB2 1GA, UK; 2 Structural Bioinformatics Group, Institute for Physiology, Charité Universitätsmedizin, Arnimallee 22, 14197 Berlin, Germany

## Abstract

**Background:**

The hydrogen bond patterns between mainchain atoms in protein structures not only give rise to regular secondary structures but also satisfy mainchain hydrogen bond potential. However, not all mainchain atoms can be satisfied through hydrogen bond interactions that arise in regular secondary structures; in some locations sidechain-to-mainchain hydrogen bonds are required to provide polar group satisfaction. Buried polar residues that are hydrogen-bonded to mainchain amide atoms tend to be highly conserved within protein families, confirming that mainchain architecture is a critical restraint on the evolution of proteins. We have investigated the stabilizing roles of buried polar sidechains on the backbones of protein structures by performing an analysis of solvent inaccessible residues that are entirely conserved within protein families and superfamilies and hydrogen bonded to an equivalent mainchain atom in each family member.

**Results:**

We show that polar and sometimes charged sidechains form hydrogen bonds to mainchain atoms in the cores of proteins in a manner that has been conserved in evolution. Although particular motifs have previously been identified where buried polar residues have conserved roles in stabilizing protein structure, for example in helix capping, we demonstrate that such interactions occur in a range of architectures and highlight those polar amino acid types that fulfil these roles. We show that these buried polar residues often span elements of secondary structure and provide stabilizing interactions of the overall protein architecture.

**Conclusions:**

Conservation of buried polar residues and the hydrogen-bond interactions that they form implies an important role for maintaining protein structure, contributing strong restraints on amino acid substitutions during divergent protein evolution. Our analysis sheds light on the important stabilizing roles of these residues in protein architecture and provides further insight into factors influencing the evolution of protein families and superfamilies.

## Background

As Pauling and Corey realised, satisfaction of hydrogen bonding potential of polypeptide mainchain functions is one of the major factors that give rise to the β-strand and α-helix [1,2]. These regular elements of secondary structure give their names to the main features of protein structure: classical β-sheets, α-helical bundles, αβ-Rossman fold, αβ-barrel and many others. Hydrogen bonding also plays important roles in the intricate and sometimes elaborate arches and turns which link α-helices and β-strands [3-5].

However, these elegant architectures still leave many mainchain functions unsatisfied in their potential to form hydrogen bonds: an early survey of hydrogen bonding in proteins revealed that ~40% of mainchain atoms do not form hydrogen bonds with other mainchain atoms [6]. In general these occur in four different circumstances:

(1) Where strands and helices terminate, requiring "capping" [6-10].

(2) Where helices and strands bulge [11,12] or bend [13,14].

(3) In polyproline or irregular, twisted strands [15,16]

(4) In arches and turns [3-5,17,18].

Water molecules or sidechains can usually satisfy the hydrogen bonding potential of mainchain functions that are at the protein surface in a variety of ways and so the residues are often substituted in evolution. However, in the smaller proportion of functions that must be satisfied from the core of the protein, this is achieved by buried sidechains of polar residues.

**Figure 1:**
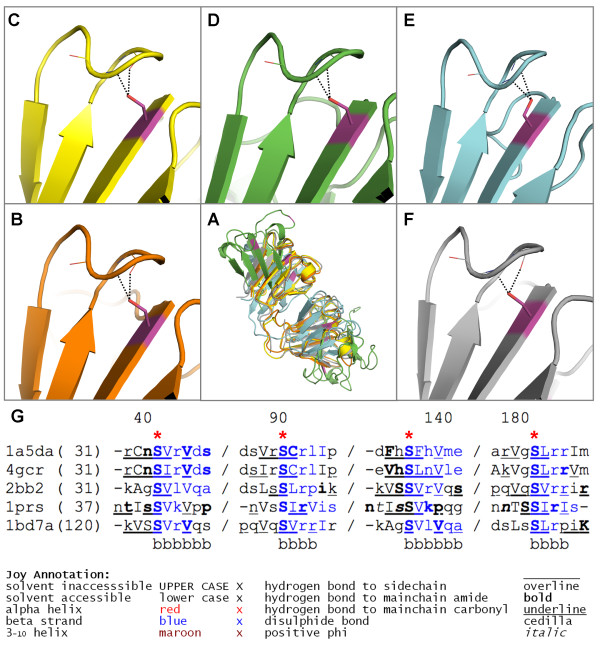
**Serine residues in the β/γ crystallin family which are conserved both in sequence and in their structural environment**. A) Superimposed cartoon representation of 5 members of the family. Four serine sidechains each form hydrogen bonds to mainchain atoms in a β-hairpin, which are conserved across the family. For clarity, one serine is shown (in magenta) in B) [PDB: 1a5d] C) [PDB: 4gcr] D) [PDB: 2bb2] E) [PDB: 1prs] and F) [PDB: 1bd7]. The conservation of these sidechain-to-mainchain interactions implies that they have an important role in the mainchain architecture of these proteins. G) Shows selected regions of a multiple sequence alignment of the β/γ crystallins containing the four conserved and buried serine residues (highlighted by red stars). The local structural environment of each residue in the alignment is displayed using JOY annotation [32]. Pictures of protein structures were produced using Pymol and clipping was used for improving figure clarity [35].

Analysis of the substitution patterns of amino acids within homologous protein families has revealed that buried polar residues that are hydrogen-bonded to mainchain amide atoms are highly conserved, more so than those polar residues forming hydrogen bonds to mainchain carbonyl atoms or other sidechains [19,20]. Furthermore, analysis of the median sequence entropy of buried amino acid residues has shown that buried polar sidechains, for which the hydrogen bond capacity is satisfied, are the most conserved amino acid residues within proteins [21]. The number of hydrogen bonds to mainchain amide groups also influences the conservation of buried satisfied polar residues, with those forming two or more being significantly more conserved than those forming only one or none [21]. Together, these results imply that the hydrogen bond functions maintained by these conserved buried polar groups have an important role in maintaining protein architecture. Figure 1 shows an example of conservation of sequence and local environment for the beta/gamma crystallin family. In the crystallins, the hydrogen bonds provided by a buried and conserved serine help to stabilize a β-hairpin structure; this is the serine that recurs in each of the four domains of β and γ crystallins and is part of the signature motif that has allowed recognition of distant homologues [22].

Previous *in silico *analyses of the stabilizing roles that polar sidechains have on the backbone of protein structures have tended to focus on a particular architectural context [13,23,24]. Bordo and Argos [25] identified recurring patterns and amino acid types involved in sidechain-to-sidechain and sidechain-to-mainchain interactions. However, the conservation of polar residues and the three-dimensional (3D) arrangements of the sidechain-to-mainchain hydrogen bonds were not considered. What then are the features of sidechain-to-mainchain hydrogen bonds formed by polar sidechains? Which amino acids are involved? What kinds of structures do these buried polar residues maintain? Are they local to a secondary structure or do they link between different helices and strands, stabilizing tertiary structure?

**Figure 2:**
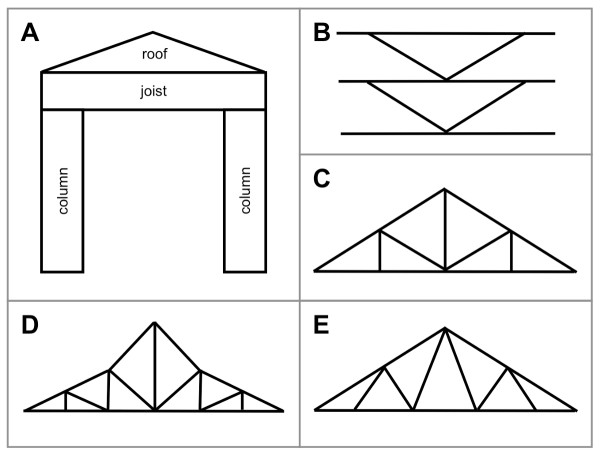
**Architectural frameworks that are similar to the stabilizing structures formed by buried and conserved polar sidechains**. Schematic diagrams of: A) a joist that spans two columns and supports the roof above; B) Vertical K-bracing which is used to provide stability to walls; C) Tri-bearing, D) Polynesian and E) double cantilever trusses are used to support structures such as roofs and bridges.

In this report we focus purely on buried polar residues that are entirely conserved within protein families and superfamilies, hydrogen bonding to a mainchain atom in each family member. We hypothesise that such buried sidechain-to-mainchain hydrogen bonds satisfy mainchain hydrogen bonding potential where secondary structures cannot be formed, and in so doing become irreplaceable elements of the overall architecture. In order to test this hypothesis we characterize the nature and tertiary structural context of these conserved and buried polar residues. We show that polar sidechains which bridge to mainchain functions in the cores of proteins have conserved tertiary structural roles in homologues. Like the elements of secondary structure, they are born of the need to satisfy hydrogen bonding but, in achieving this, they become key, conserved structural features of many well-known protein architectures. Some are joists or braces, spanning the helices and strands, while others form truss-like structures that support complex loop structures (Figure 2).

## Results and Discussion

### Buried polar residues stabilizing protein architecture through conserved interactions

In HOMSTRAD [26], a database of structurally aligned families, 143 families have five or more members with high resolution structures, 131 of which are non-redundant i.e. their sequence alignments do not overlap - see Additional file 1, Table S1. Of these, 65 have conserved and buried polar residues, providing a total of 233 alignment positions where the equivalent residue in each structure forms a hydrogen bond through its sidechain to a mainchain atom - see Additional file 2, Table S2. The frequency of occurrence for the polar amino acids at these 233 alignment positions are shown in Table 1. We have examined the propensity with which such conserved and buried polar residues participate in various architectural motifs - shown in Table 2. We have focused on interactions that are conserved in families, on the assumption that these have had a selective advantage and may teach us about important factors that determine protein architectures.

**Table 1:** Shows the frequency of occurrence for each polar amino acid in the 233 conserved positions.

Amino Acid	Number
Arg	98
Asn	133
Asp	313
Cys	161
Gln	68
Glu	86
His	121
Lys	39
Ser	155
Thr	174
Trp	92
Tyr	144

The analysis only considered conserved positions containing polar amino acids therefore the frequency of occurrence for non-polar amino acids is zero.

**Table 2:** Propensity of polar residues forming sidechain hydrogen bonds to mainchain atoms in various architectural contexts.

	Propensity of polar residues forming sidechain hydrogen bonds to mainchain atoms																			
	
Amino Acid	Within helix N-termini		Within helix C-termini		Within edge strands		From edge strands		Within central strands		From central strands		Within 3_10 _helices		Within β-hairpins		Within polyproline helices		Within coil regions	
	
	Con	All	Con	All	Con	All	Con	All	Con	All	Con	All	Con	All	Con	All	Con	All	Con	All
Arg	1.05	0.46	14.38	6.44	3.17	3.29	1.71	3.25	0.34	1.73	3.07	3.42	3.02	3.52	1.48	2.69	6.11	3.70	2.48	3.52
Asn	1.30	2.88	1.14	1.84	3.12	3.20	3.20	2.00	2.44	2.44	3.06	2.30	1.58	2.41	1.13	3.05	2.54	2.43	1.85	2.57
Asp	4.84	3.04	0.61	0.54	2.65	1.20	2.27	2.06	0.97	1.44	2.42	1.46	3.04	2.28	6.37	2.97	1.74	1.43	5.20	2.52
Cys	9.86	2.20	4.52	1.40	3.83	2.66	0.93	2.22	5.09	4.06	0.39	2.53	8.99	2.59	4.51	1.70	1.55	0.84	5.94	1.97
Gln	0.11	1.22	0.44	1.80	2.33	2.98	6.19	2.23	4.87	1.86	1.67	2.10	0.96	1.69	1.04	1.80	1.55	3.06	0.52	1.96
Glu	1.94	1.40	0.00	0.06	0.00	0.82	0.00	0.64	0.57	0.48	2.35	0.78	0.81	0.79	0.08	0.93	0.80	1.19	0.90	0.99
His	2.37	1.13	0.89	2.50	0.97	2.24	4.08	2.14	0.82	2.08	1.81	2.22	1.45	2.62	1.90	1.89	4.71	2.38	2.66	2.08
Lys	0.00	0.13	0.60	1.99	0.00	1.05	0.00	0.88	1.25	0.51	0.00	1.12	0.15	1.18	0.00	0.67	0.40	1.00	0.13	0.97
Ser	1.53	3.43	0.65	2.15	0.47	1.92	2.61	2.01	2.32	3.22	2.46	1.75	1.09	2.01	3.58	2.23	0.76	2.03	0.90	1.95
Thr	1.53	3.64	0.70	2.05	2.91	1.81	0.68	2.25	3.43	3.29	0.48	2.37	0.65	1.88	0.75	2.23	0.62	1.88	1.19	1.81
Trp	0.81	0.23	0.36	1.28	2.06	1.31	6.53	2.01	1.84	1.83	1.84	1.83	3.41	1.23	3.88	1.40	2.93	1.25	3.58	1.30
Tyr	1.06	0.68	0.56	1.23	4.04	1.77	0.30	2.37	1.03	1.26	3.14	2.37	4.20	1.37	0.07	0.91	3.12	1.84	1.60	1.39

Columns headed "Col" display the propensities of conserved buried polar residues.

Columns headed "All" display the propensities of all polar residues forming the indicated interaction (regardless of solvent accessibility or conservation).

### Interactions with the N-terminal regions of α-helices

For conserved and buried polar residues making hydrogen bonds to mainchain NH functions in the N-terminal regions of α-helices, cysteine has the highest propensity to form such interactions, followed by negatively charged aspartate, histidine and glutamate (Table 2 and see Additional file 3, Figure S1A - grey bars); surprisingly, neutral residues such as serine, threonine and asparagine have higher propensities when solvent accessible positions are considered (Table 2 and see Additional file 3, Figure S1A - white bars) [8,27,28]. This may reflect the importance of the charged hydrogen bond in regions of low dielectric strength, as well as its interaction with the helix dipole [29].

**Figure 3:**
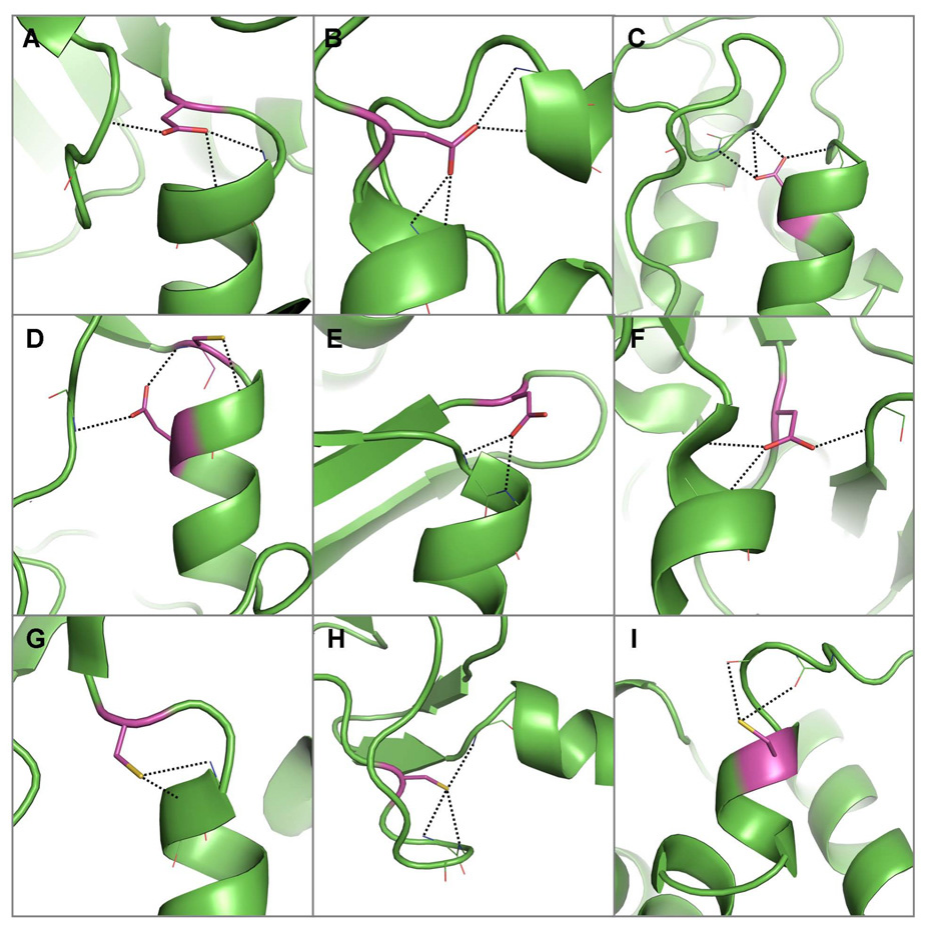
**Examples of hydrogen bond interactions from conserved, buried polar residues that involve N-terminal regions of α-helices**. Representative structures were chosen for each family based on resolution; residues are coloured by atom type with buried, conserved polar residues shown in magenta. Hydrogen bonds are shown in black. Two examples of aspartates that hydrogen bond forward to N-termini residues in A) the glyceraldehyde 3-phosphate dehydrogenase family [PDB: 1gd1] and B) the beta-lactamase family [PDB: 1btl]. The aspartate in B) also forms distant interactions to another helix N-terminus. Two examples of aspartates hydrogen bonding back to N-terminal residues as well as to coils in the C) alcohol dehydrogenases [PDB: 2ohx] and D) matrix metalloproteinases [PDB: 1hfc]. The latter panel also displays an example of a cysteine hydrogen-bonding forward to an N-terminal residue. Two examples of aspartates forming distant hydrogen bonds to N-terminal residues in E) bacterial serine proteinases [PDB: 2sga] and F) chalcone and stilbene synthases [PDB: 1hnj]. G) Cysteine residue that hydrogen bonds forward to an N-terminal region in cytochrome P450 s [PDB: 1jfb]. H) Cysteine in zinc binding domain in Lin-11, Isl-1 and Mec-3 [PDB: 1ctl] that forms distant hydrogen bonds to an N-terminal residue. I) Cysteine hydrogen bonding back to N-terminal residues in the alcohol dehydrogenase family [PDB: 2ohx]. Pictures of all polar sidechain examples were produced using Pymol and clipping was used for improving figure clarity [35].

Local capping effects of buried aspartates occurring either upstream (Figure 3A-B) or downstream (Figure 3C-D) from their hydrogen bonded partner have been well described, but less attention has been paid to aspartates that are hydrogen bonded to the N-terminal residue of a helix via a distant interaction (Figure 3B, E-F), providing structures that often resemble joists. Similar hydrogen bonded interactions are made by cysteines with N-terminal residues, except that cysteine mostly occurs upstream (Figure 3D, G) or interacts distantly (Figure 3H) and is rarely observed to occur downstream from the N-terminal residue (Figure 3I).

### Interactions with the C-terminal regions of α-helices

**Figure 4:**
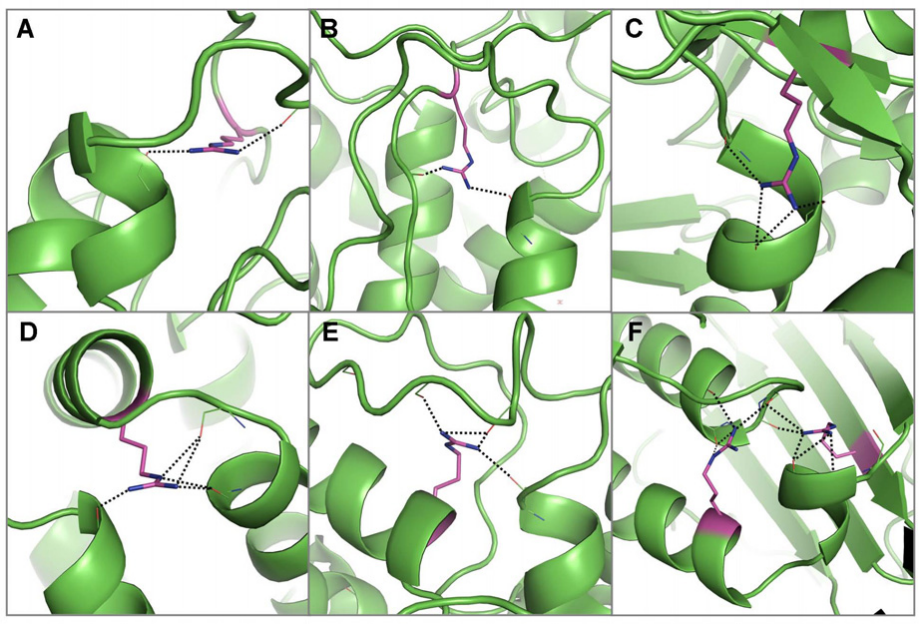
**Examples of hydrogen bond interactions from conserved, buried arginine residues to mainchain atoms in α-helix C-terminal regions**. Representative structures were chosen for each family based on resolution; residues are coloured by atom type with buried, conserved polar residues shown in magenta. Hydrogen bonds are shown in black. Two examples of arginine residues forming hydrogen bonds to C-terminal regions of α-helices from coil regions in A) the eukaryotic-type carbonic anhydrase family [PDB: 1koq] and B) the peroxidise family [PDB: 2cyp]. C) An arginine in a β-sheet forms two hydrogen bonds to a C-terminal region in the chalcone and stilbene synthases [PDB: 1i88]. D) An arginine in the N-terminal region of an α-helix forms hydrogen bonds to C-terminal regions of two helices in the annexin family [PDB: 1axn]. Two examples of arginine residues in C-terminal regions of α-helices that form hydrogen bonds to C-terminal regions in other helices in E) the cytochrome P450 s [PDB: 1jfb] and F) the cyclodextrin glycosyltransferases [PDB: 1qhp]. In the latter case a second arginine within a β-sheet also interacts with C-terminal residues of a third short helix.

In a similar way to the aspartates that interact with N-terminal regions of α-helices, the charged residue, arginine, has the highest propensity to form capping interactions that are both conserved and buried at the C-termini of α-helices, while at the same time compensating for the helix dipole (Table 2 and see Additional file 3, Figure S1B - grey bars). Interestingly, all conserved, buried arginine residues that interact with C-terminal residues do so distantly (Figure 4), often with the arginine itself also being found within a capping region of a different helix (Figure 4D-F). This feature often occurs when the C-termini of multiple helices are aligned (Figure 4D-F), no doubt providing favourable interactions with the negative helix dipoles by helping to offset charge repulsion between two or more helix C-termini.

### Interactions with edge strands

**Figure 5:**
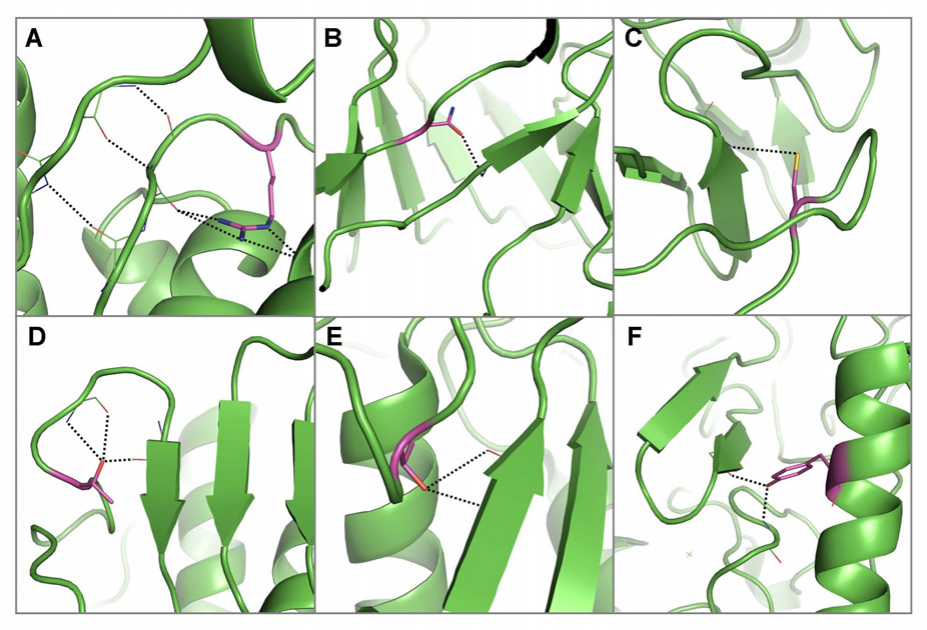
**Examples of hydrogen bond interactions from conserved, buried residues to mainchain atoms in edge strands**. Representative structures were chosen for each family based on resolution; residues are coloured by atom type with buried, conserved polar residues shown in magenta. Hydrogen bonds are shown in black. A) An arginine in the peroxidases which forms hydrogen bonds to an edge strand [PDB: 1qpa]. B) An example of asparagine forming a hydrogen bond to a strand in a β-barrel (classified as edge strands) in the xylose isomerise family [PDB: 1bxb]. C) A cysteine forms a hydrogen bond with a strand in a β-sheet in the Zinc-binding domain present in Lin-11, Isl-1, Mec-3 protein family [PDB: 1a7i]. Two examples of threonines forming hydrogen bonds to edge strands in D) the pyridine nucleotide-disulphide oxidoreductases (class 1) [PDB: 3grs] and E) the aldehyde oxidase and xanthine dehydrogenase (domains 3&4) family [PDB: 1n62]. F) A tyrosine in cyclodextrin glycosyltransferases forms a hydrogen bond to an edge strand [PDB: 1qhp].

The polar amino acids with the highest propensities for interacting with edge strands are arginine, asparagine, glutamine and cysteine (Table 2 and see Additional file 3, Figure S2A - white bars). However, of conserved, buried polar residues making hydrogen bonds to mainchain atoms in edge strands, tyrosine has the highest propensity to form such an interaction, followed by cysteine, arginine, asparagine and threonine, although the propensities are rather low (Table 2 and see Additional file 3, Figure S2A - grey bars). Without the hydrogen bonds from buried, conserved sidechains, these mainchain atoms in edge strands would otherwise form no hydrogen bonds (Figure 5). They include strands in β-barrels that are staggered and have no neighbouring strands (Figure 5B-C).

### Interactions from within edge strands

**Figure 6:**
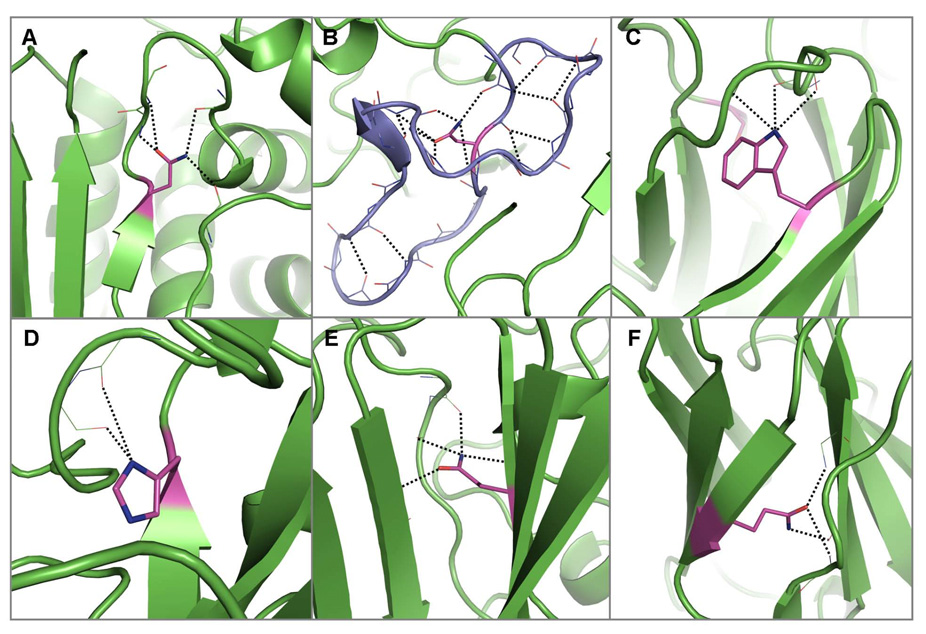
**Examples of hydrogen bond interactions from conserved, buried residues within edge strands to mainchain atoms**. Representative structures were chosen for each family based on resolution; residues are coloured by atom type with buried, conserved polar residues shown in magenta. Hydrogen bonds are shown in black. A) An asparagine forms hydrogen bonds to mainchain atoms in a type IV β-turn in aspartate/ornithine carbamoyltransferases [PDB: 1oth]. Within the cyclodextrin glycosyltransferases [PDB: 1qhp] B) an asparagine forms hydrogen bonds to mainchain atoms within a complicated β-hairpin structure (strands and hairpin shown in purple). C) A tryptophan forms hydrogen bonds to a turn joining two β-strands in the cyclodextrin glycosyltransferases [PDB: 1qhp]. D) Histidine forming hydrogen bonds to a 3_10 _helix in the Cu/Zn superoxide dismutase family [PDB: 2aps]. Two examples of glutamine residues forming hydrogen bonds to mainchain atoms in another strand within a β-sandwich in E) the picornavirus coat protein family (also forms hydrogen bonds to mainchain atoms within a 3_10 _helix and coil region) [PDB: 2plv] and F) the immunoglobulin domain (V set) light chain family [PDB: 6fab].

Arginine, followed by tyrosine and threonine (Table 2 and see Additional file 3, Figure S2B - white bars) have the highest propensity to form hydrogen bonds to mainchains within edge strands. However, amongst conserved and buried residues within edge strands, tryptophan has the highest propensity, followed by glutamine, histidine and asparagine (Table 2 and see Additional file 3, Figure S2B - grey bars). Asparagine and tryptophan often interact (locally) with regions connecting regular secondary structures e.g. β-turns and β-hairpins (Figure 6A-C), while glutamine can bridge the gap between two strands in β-barrel structures (Figure 6E-F).

### Interactions with centre strands

**Figure 7:**
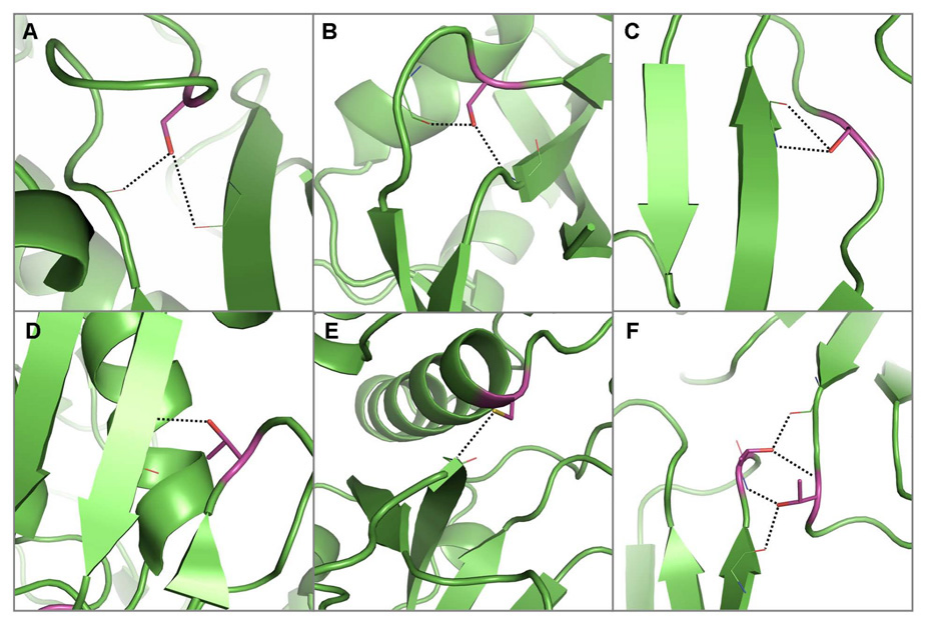
**Examples of hydrogen bond interactions from conserved, buried residues to mainchain atoms in centre strands**. Representative structures were chosen for each family based on resolution; residues are coloured by atom type with buried, conserved polar residues shown in magenta. Hydrogen bonds are shown in black. A) A serine residue within a coil forming hydrogen bonds to two strands that have deviated away from each other in the haloperoxidases [PDB: 1b6g]. Examples of polar residues that form hydrogen bonds to an adjacent strand that extends further than its neighbour, including serines in B) the pancreatic ribonuclease family [PDB: 7rsa] and C) the cyclodextrin glycosyltransferases [PDB: 1qhp], D) a threonine in the aldehyde oxide and xanthine dehydrogenases (domains 1&2) [PDB: 1fo4] and E) a cysteine in the papain family cysteine proteinase [PDB: 1mem]. F) Two threonines that form hydrogen bonds to each other's mainchain amide atoms as well as atoms within strands (one central, one edge) in the aspartic proteinases [PDB: 3app].

The mainchains in centre strands are sometimes unable to form hydrogen bonds with the neighbouring strand. Examples include where two strands curve away from each other (Figure 7A), where the neighbouring strand is shorter than the central strand in question (Figure 7B-E), or where the mainchain atom is at the terminus of a strand (Figure 7B,C,E) or part of a β-barrel (Figure 6E). These mainchain functions are often satisfied by sidechain hydrogen bonds. Of polar residues that are conserved and buried and carrying out this role, cysteine, glutamine, threonine, asparagine and serine have the highest propensity to form such interactions (Table 2 and see Additional file 3, Figure S2C - grey bars). In some cases the sidechains act as "braces"; for example, the threonines of the conserved aspartic proteinases Asp-Thr-Gly triplet, where the strands diverge after the threonine on either side of the pseudo dyad in the eukaryotic enzymes or the dyad of the dimeric retroviral enzymes (Figure 7F).

### Interactions from within centre strands

Of conserved, buried polar residues within centre strands forming hydrogen bonds to mainchain atoms, tyrosine has the highest propensity to form such interactions, followed closely by arginine, asparagine, serine, aspartate and glutamate (Table 2 and see Additional file 3, Figure S2D - grey bars). We see a different pattern however when we consider all polar amino acids in centre strands that form hydrogen bonds to mainchain atoms - arginine has the highest propensity to form this type of interaction followed by cysteine, tyrosine, threonine and asparagine (Table 2 and see Additional file 3, Figure S2D - white bars). Asparagine, aspartate, glutamate, serine and tyrosine are more commonly found to form hydrogen bonds to mainchain atoms from within edge strands when conservation and solvent accessibility are considered whereas threonine and cysteine are less common.

**Figure 8:**
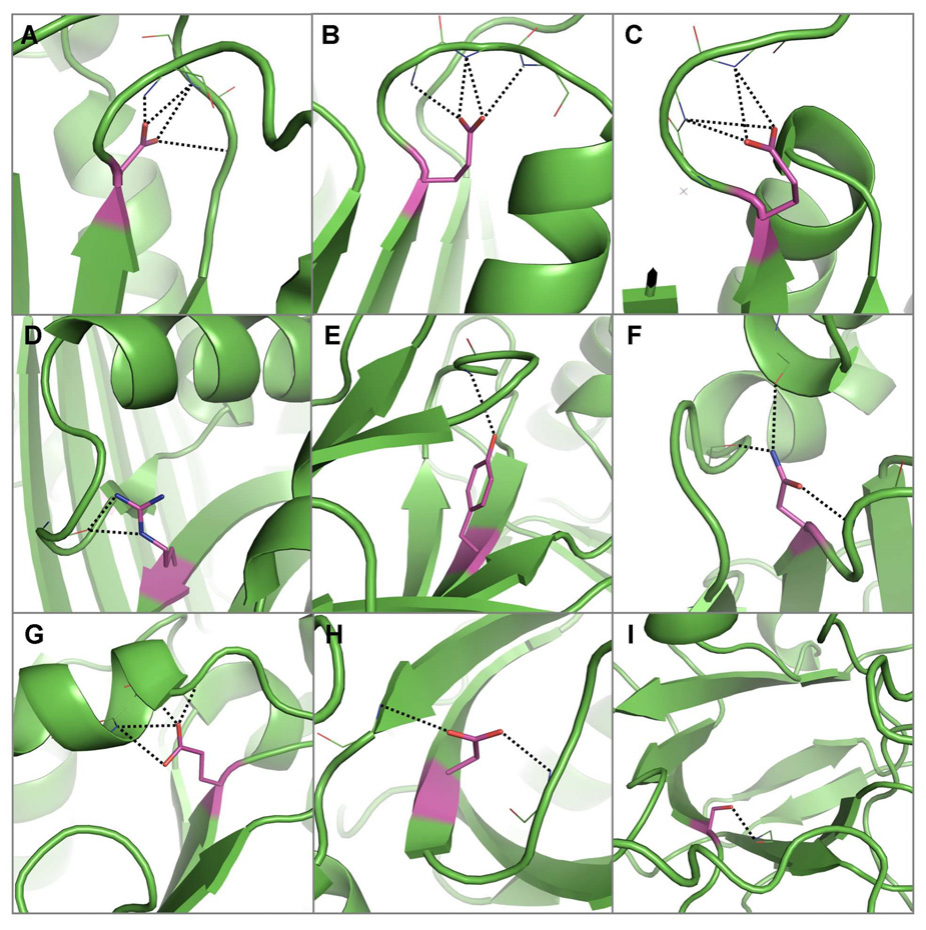
**Examples of hydrogen bond interactions from conserved, buried residues in centre strands to mainchain atoms**. Representative structures were chosen for each family based on resolution; residues are coloured by atom type with buried, conserved polar residues shown in magenta. Hydrogen bonds are shown in black. Examples of coils that are supported by hydrogen bonds from polar residues at the end of central strands, including A) an aspartate in the aldehyde oxidase and xanthine dehydrogenases (domains 3&4) [PDB: 1n62], two glutamates in B) the alcohol dehydrogenases [PDB: 2ohx] and C) the isocitrate and isopropylmalate dehydrogenases [PDB: 1cnz] and D) an arginine in the serine proteinase inhibitor family [PDB: 1hle]. E) A tyrosine residue in the pancreatic lipase family forms a hydrogen bond with a type IV β-turn [PDB: 1bu8]. Two cases where mainchain atoms in helices are satisfied by hydrogen bonds from F) an asparagine in eukaryotic-type carbonic anhydrases [PDB: 1koq] and G) a glutamate in the NADH ubiquinone oxidoreductases. Examples of mainchain atoms in β-barrel strands that are satisfied by hydrogen bonds from H) an aspartate in PDZ domain proteins [PDB code 1be9] and I) a serine in the aldo/keto reductases [PDB: 1ads].

The conserved, buried polar residues within centre strands that form hydrogen bonds to mainchain atoms tend to occur at the termini of strands more often than in the middle of the strand (Figure 8). They often interact with coils (Figure 8A-D), β-turns (Figure 8E) and polyproline, forming truss-like structures that support the coil-like regions they are interacting with. Others are observed to interact with helix capping regions (Figure 8F-G) and neighbouring strands in β-barrels, forming structures that resemble joists (Figure 8H-I).

### Interactions to residues within 3_10 _helices

**Figure 9:**
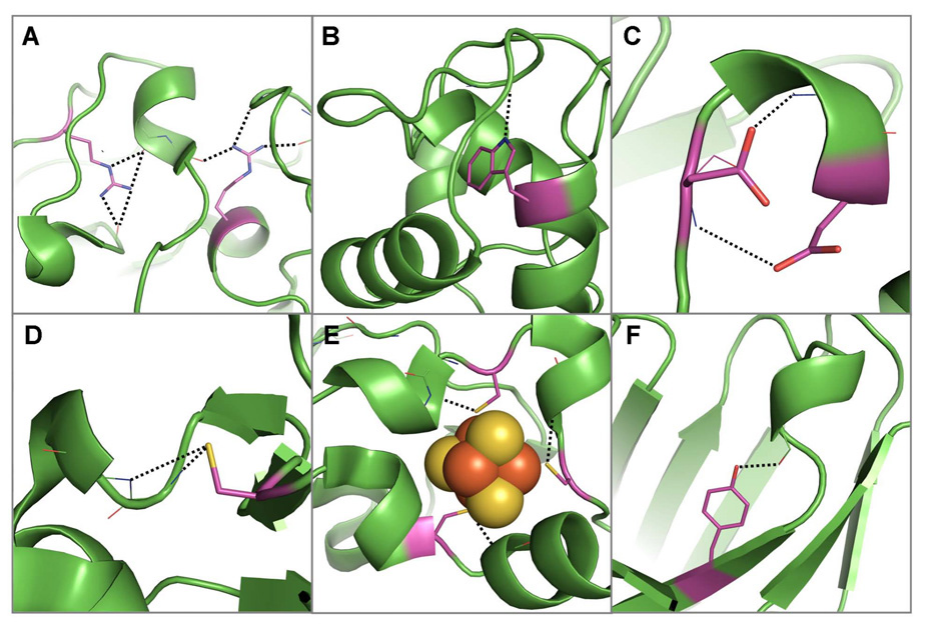
**Examples of hydrogen bond interactions from conserved, buried residues to mainchain atoms in 3_10 _helices**. Representative structures were chosen for each family based on resolution; residues are coloured by atom type with buried, polar residues shown in magenta. Hydrogen bonds are shown in black. A) Two arginines in the cyclodextrin glycosyltransferases family which hydrogen bond to two 3_10 _helices [PDB: 1d3c]. B) A tryptophan that forms a hydrogen bond to a 3_10 _helix in the papain family cysteine proteinases [PDB 1mem]. C) Two aspartates that form a hydrogen bond to each other's respective mainchain amide atom group in a 3_10 _helix in the pancreatic lipases [PDB: 1bu8]. D) A cysteine forms a hydrogen bond with a 3_10 _helix in the aldehyde oxidase and xanthine dehydrogenase family (domains 1&2) [PDB: 1n62] E) Three cysteines that form a complex with an iron sulphate (4Fe-4S) cluster also form hydrogen bonds to 3_10 _helices that form the binding site [PDB: 1e3d]. F) A tyrosine within a central strand forms a hydrogen bond to a mainchain carbonyl within a 3_10 _helix in the immunoglobulin domain (V set) family [PDB 2rhe].

Cysteine has the highest propensity of buried, conserved polar residues to form hydrogen bonds to mainchain atoms in 3_10 _helices, followed by tyrosine, tryptophan, aspartate and arginine (Table 2 and see Additional file 3, Figure S3 - grey bars). This differs to all polar amino acids interacting with 3_10 _helices where arginine, histidine, cysteine and asparagine have the highest propensities (Table 2 and see Additional file 3, Figure S3 - white bars). There is less of a clear preference for the 3_10 _helices to hydrogen bond with particular polar sidechains than in α-helices, probably due to the greater plasticity in these helices, which usually comprise only two or three turns (Figure 9).

### Interactions with beta hairpins

In β-hairpins, mainchain atoms that are hydrogen-bonded to conserved and buried sidechains have a high propensity to interact with aspartate, cysteine, tryptophan and serine (Table 2 and see Additional file 3, Figure S4 - grey bars). We see a similar pattern when we consider all polar amino acids forming hydrogen bonds to mainchain atoms in β-hairpins; asparagine has the highest propensity to form this type of interaction followed by aspartate, arginine, serine and threonine (Table 2 and see Additional file 3, Figure S4 - white bars). Therefore, although asparagine, arginine and threonine often form hydrogen bonds to mainchain atoms within β-hairpins, these interactions tend not to be conserved in buried positions.

**Figure 10:**
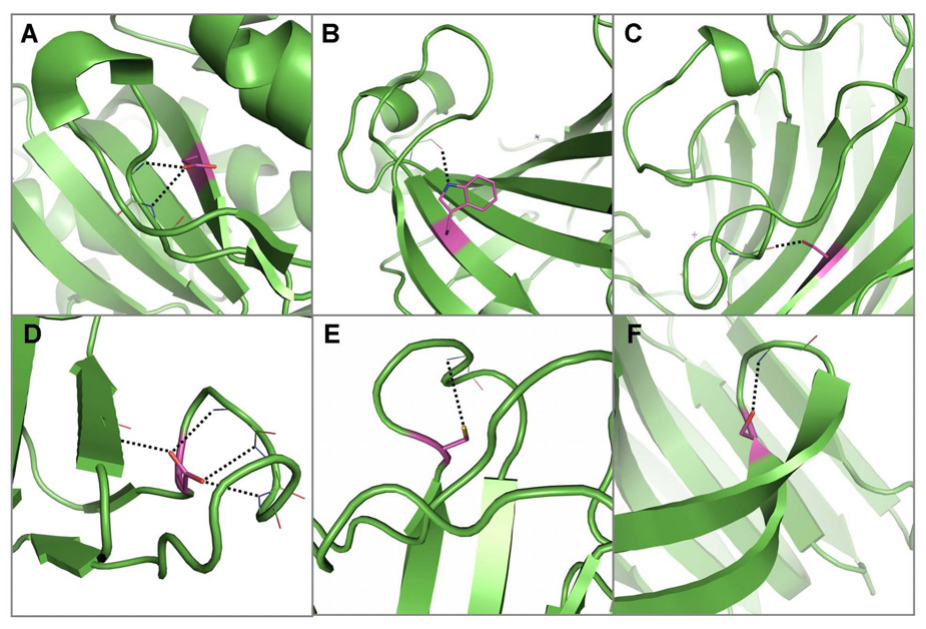
**Examples of hydrogen bond interactions from conserved, buried residues to mainchain atoms in β-hairpins**. Representative structures were chosen for each family based on resolution; residues are coloured by atom type with buried, conserved polar residues shown in magenta. Hydrogen bonds are shown in black. Examples of polar sidechains interacting distantly to form hydrogen bonds with mainchain amide groups in β-hairpins including, A) an aspartate in the ribulose bisphosphate carboxylases [PDB: 1gk8], B) a tryptophan in the eukaryotic-type carbonic anhydrases [PDB: 1ca2] and C) a serine in the legume lectins [PDB: 2ltn]. D) An aspartate in the cyclodextrin glycosyltransferases forms a hydrogen bond to an edge strand as well as forming local interactions to a type 12:12 β-hairpin [PDB: 1qhp]. Two examples of polar residues forming hydrogen bonds with mainchain atoms in β-hairpins via a local interaction including, E) a cysteine in the azurin/plastocyanin family and F) a serine in the glycosyl hydrolase family 11 [PDB: 1xnb].

The conserved buried polar residues that form hydrogen bonds to mainchain atoms in β-hairpins almost always interact distantly with mainchain atoms that would otherwise form no hydrogen bonds (Figure 10). Some of the β-hairpin structures are extremely long and complex (Figure 10A-C).

### Interactions with polyproline

From the set of conserved, buried polar residues hydrogen-bonded to mainchain atoms of polyproline-type helices, arginine is most common, followed by histidine, tyrosine and tryptophan (Table 2 and see Additional file 3, Figure S5 - grey bars). Arginine also has the highest propensity to form this interaction when we consider all residues forming this type of interaction, followed by glutamine, asparagine and histidine (Table 2 and see Additional file 3, Figure S5 - white bars). A similar result has previously been observed where hydrogen bonds from sidechains to mainchains in polyproline were most frequently formed by arginine followed by glutamine, asparagine, serine and threonine [15].

**Figure 11:**
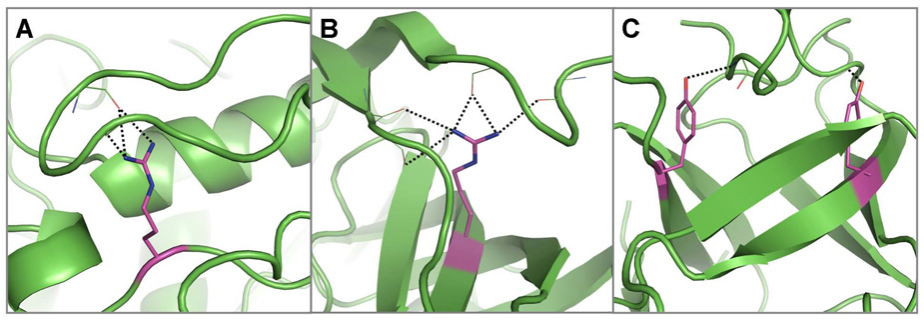
**Examples of hydrogen bond interactions from conserved, buried residues to mainchain atoms in polyproline helices**. Representative structures were chosen for each family based on resolution; residues are coloured by atom type with buried, conserved polar residues shown in magenta. Hydrogen bonds are shown in black. Two examples of arginines forming hydrogen bonds to polyproline helices in A) the α/β hydrolases [PDB: 2bce] and B) the alcohol dehydrogenases - polyproline interaction on the right [PDB: 2ohx]. C) Two tyrosines in the eukaryotic serine proteinases [PDB: 1avw] forming hydrogen bonds to polyproline helices which form an interaction site with trypsin.

Polyproline helices are extended and most often occur on the surface of proteins [30]; it is therefore not surprising that the conserved, buried residues that form hydrogen bonds come from a residue distant in the sequence. Typical examples are shown in Figures 11A and 11B from the α/β hydrolases and the alcohol dehydrogenases, respectively. In such a mode, the polar residues form truss-like structures that help to stabilize the irregular polyproline helices.

### Interactions with coil regions

Cysteine and aspartate clearly have the highest propensity to form hydrogen bonds to coil regions out of buried conserved polar residues (Table 2 and see Additional file 3, Figure S6 - grey bars). However, arginine has the highest propensity to perform this role when all positions are considered, followed by asparagine and aspartate (Table 2 and see Additional file 3, Figure S6 - white bars). A previous analysis of intra-coil sidechain-to-mainchain hydrogen bonds revealed that aspartate, serine, asparagine and threonine are the polar residues that most commonly form this type of interaction, with 80% of these cases being at solvent-exposed sites [25].

**Figure 12:**
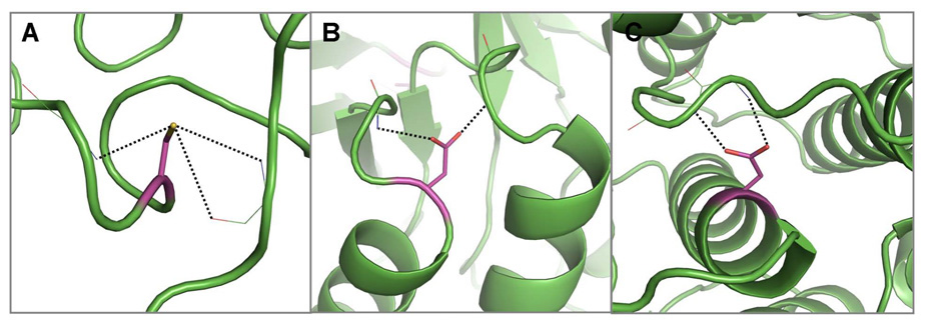
**Examples of hydrogen bond interactions from conserved, buried residues to mainchain atoms in coils**. Representative structures were chosen for each family based on resolution; residues are coloured by atom type with buried, conserved polar residues shown in magenta. Hydrogen bonds are shown in black. Three examples of polar residues forming hydrogen bonds to coils including, A) a cysteine in the high potential iron-sulphur protein family [PDB: 1isu], two asparates in B) the glycosyl hydrolase family 10 [PDB: 1tax] and C) the serine/threonine protein kinases [PDB: 1hcl].

Polar sidechains frequently form hydrogen bonds to coil regions, often in very elaborate loop structures that form extended turns and arches [3-5] (Figures 3A,C-D,H; 4A-B,E; 5D,F; 6A,C,D; 8A-F). However, there are also instances where the conserved and buried residues only form hydrogen bonds with mainchain atoms in coil regions, indicating that stabilization of these irregular regions by polar sidechains is important enough for them to be conserved during evolution (Figure 12).

## Conclusions

We have previously demonstrated that buried polar residues, although small in number, tend to be more conserved when their hydrogen-bonding potential is satisfied or where they form hydrogen bonds to mainchain atoms [21]. Conservation of these residues and the interactions that they form implies that they are important for maintaining protein structure and hence provide restraints on amino acid substitutions during divergent evolution. We have shown that conserved, buried polar residues have conserved roles in stabilizing the tertiary structure of proteins by forming hydrogen bonds to mainchain atoms. The conservation of these sidechain-to-mainchain hydrogen bonds implies that mainchain architecture is a crucial restraint on the evolution of proteins and that the interactions are retained as an essential part of the protein fold. The structural motifs that we have examined have been shown to have particular propensities for polar residues which form hydrogen bonds with mainchain atoms. Although local sidechain-to-mainchain interactions have been the focus of most previous studies, the propensity for sidechain-to-mainchain hydrogen bond formation is often met by distant interaction. For example, we observe that arginine frequently caps the C-termini of α-helices through a distant interaction. We have shown that buried polar residues maintain 3D relationships between secondary structures where mainchain-to-mainchain hydrogen bonds cannot play a role and that similar stabilizing structures recur in different architectures. The key roles of these stabilizing interactions in maintaining protein structures have been previously demonstrated in a few cases, for example in the tyrosine corner [31], but we have shown here that there are many others important for maintaining protein stability.

Although it is generally unfavourable to bury hydrophilic amino acids in the core of proteins, this is counterbalanced by the need to satisfy mainchain atom hydrogen-bond potential. The interactions that the polar residues form when providing these supporting roles are often quite complex and can be thought of as analogous to features in our own built 3D environment. Many form joists, bridging between the elements of secondary structure (for example, Figures 3B, 4D-F, 5B-C, 7A-E), analogous to those that bridge columns and support structures above them in man-made buildings (Figure 2A). Other sidechains act as braces, tethering two strands at the point at which they diverge (Figure 7F and Figure 2B). Buried hydrogen bonded polar sidechains often maintain triangulated structures, supporting distorted helices and complex loop structures (Figures 3I, 6A,C, 8A-C, 11A-B): these provide a striking parallel with the trusses supporting the roofs of buildings (Figure 2C-E). Remarkably, these structural features have been highly conserved in their respective architectural histories, despite the variation in surface structures. Both are hidden from view and remain unappreciated, except by the cognoscenti. We hope that this paper will help bring understanding of these important structural features of protein architecture to a wider audience.

## Methods

### Dataset

Protein families containing five or more members were selected from HOMSTRAD where the family alignment contained a conserved, buried polar residue and where the sidechain of the polar residue forms a hydrogen bond to a mainchain atom in each family member. The JOY[32] alignment of each family within HOMSTRAD was used to identify families that met these criteria. JOY's default relative accessibility cut-off (7% or less) was used to define solvent inaccessible (buried) residues. In order to avoid redundancy, where protein families overlapped, the family with the highest sequence coverage was chosen for the analysis.

### Identification of hydrogen bond partners

Hydrogen bond partner(s) to the conserved, buried polar residues were identified using the program, HBOND (J. Overington, unpublished). HBOND identifies all possible hydrogen bonds based on a distance criterion (3.5Å between donor and acceptor).

### Identification of structural motifs

We used the program, PROMOTIF, to identify the structural context of the conserved polar residues and their interaction partners [33]. The following motifs were identified:

1. α-helices (N-terminal and C-terminal residues were identified based on the following positional criteria: N-(N+1) to N-(N+3) for N-terminal residues and N-3 to N+1 for C-terminal residues (where N is the length of the helix).

2. 3_10 _helices

3. β-strands - edge strands were distinguished from centre strands by referring to the number of hydrogen bonding partner strands. Strands defined as having >1 hydrogen bonding partner strand were defined as centre and all others as edge.

4. β-hairpins

5. Coil regions

We also identified polyproline helices using the program SEGNO[34].

### Calculation of residue propensities

The propensity of a particular residue type *x *to form hydrogen bonds to mainchain atoms in a particular architectural context *P*_*arch *_was calculated using the following equation:

where *n*_*arch*(*x*) _is the number of residues of type *x *forming hydrogen bonds to mainchain atoms in a particular architectural context, *N*_(*x *) _is the number of residues of type *x *in the dataset of 131 families, *n*_*arch*(*total*) _is the total number of residues forming hydrogen bonds to mainchain atoms in a particular architectural context and *N*_(*total*) _is the total number of residues in the dataset of 131 families.

Propensities were calculated for:

(i) Polar residues which are entirely conserved, buried in each family member and forming a hydrogen bond to a mainchain atom group in each family member. These numbers were therefore derived from the 233 alignment positions identified in the 66 families.

(ii) All polar residues in the 131 family set, regardless of solvent accessibility and conservation but where the polar residue forms a hydrogen bond to a mainchain atom group.

## Authors' contributions

CLW participated in the design of the study, performed the computational experiments, analysed the data and drafted the manuscript. TLB conceived of the study, participated in its design and refined the manuscript. Both authors read and approved the final manuscript.

## Supplementary Material

Additional file 1 **Table of the 131 non-redundant families which were used in the analysis**. Click here for file

Additional file 2 **Table of the families and their members that were used in the analysis**. Click here for file

Additional file 3 **Figures S1 to S6 show the propensity of polar amino acids to form hydrogen bonds to mainchain atoms in the various architectural contexts analysed**. Click here for file

## References

[B1] Pauling L Corey RB Configurations of Polypeptide Chains With Favored Orientations Around Single Bonds: Two New Pleated Sheets Proc Natl Acad Sci USA 1951 37 11 729 740 10.1073/pnas.37.11.729 16578412 PMC1063460

[B2] Pauling L Corey RB Branson HR The structure of proteins; two hydrogen-bonded helical configurations of the polypeptide chain Proc Natl Acad Sci USA 1951 37 4 205 211 10.1073/pnas.37.4.205 14816373 PMC1063337

[B3] Hutchinson EG Thornton JM A revised set of potentials for beta-turn formation in proteins Protein Sci 1994 3 12 2207 2216 10.1002/pro.5560031206 7756980 PMC2142776

[B4] Wilmot CM Thornton JM Analysis and prediction of the different types of beta-turn in proteins J Mol Biol 1988 203 1 221 232 10.1016/0022-2836(88)90103-9 3184187

[B5] Sibanda BL Blundell TL Thornton JM Conformation of beta-hairpins in protein structures. A systematic classification with applications to modelling by homology, electron density fitting and protein engineering J Mol Biol 1989 206 4 759 777 10.1016/0022-2836(89)90583-4 2500530

[B6] Baker EN Hubbard RE Hydrogen bonding in globular proteins Prog Biophys Mol Biol 1984 44 2 97 179 10.1016/0079-6107(84)90007-5 6385134

[B7] Presta LG Rose GD Helix signals in proteins Science 1988 240 4859 1632 1641 10.1126/science.2837824 2837824

[B8] Richardson JS Richardson DC Amino acid preferences for specific locations at the ends of alpha helices Science 1988 240 4859 1648 1652 10.1126/science.3381086 3381086

[B9] Wan WY Milner-White EJ A recurring two-hydrogen-bond motif incorporating a serine or threonine residue is found both at alpha-helical N termini and in other situations J Mol Biol 1999 286 5 1651 1662 10.1006/jmbi.1999.2551 10064721

[B10] Wan WY Milner-White EJ A natural grouping of motifs with an aspartate or asparagine residue forming two hydrogen bonds to residues ahead in sequence: their occurrence at alpha-helical N termini and in other situations J Mol Biol 1999 286 5 1633 1649 10.1006/jmbi.1999.2552 10064720

[B11] Chan AWE Hutchinson EG Thornton JM Identification, classification, and analysis of beta-bulges in proteins Protein Sci 1993 2 1574 1590 10.1002/pro.5560021004 8251933 PMC2142268

[B12] Richardson JS Getzoff ED Richardson DC The beta bulge: a common small unit of nonrepetitive protein structure Proc Natl Acad Sci USA 1978 75 6 2574 2578 10.1073/pnas.75.6.2574 275827 PMC392604

[B13] Eswar N Ramakrishnan C Secondary structures without backbone: an analysis of backbone mimicry by polar side chains in protein structures Protein Eng 1999 12 6 447 455 10.1093/protein/12.6.447 10388841

[B14] Barlow DJ Thornton JM Helix geometry in proteins J Mol Biol 1988 201 3 601 619 10.1016/0022-2836(88)90641-9 3418712

[B15] Cubellis MV Caillez F Blundell TL Lovell SC Properties of polyproline II, a secondary structure element implicated in protein-protein interactions Proteins 2005 58 4 880 892 10.1002/prot.20327 15657931

[B16] Stapley BJ Creamer TP A survey of left-handed polyproline II helices Protein Sci 1999 8 3 587 595 10091661 10.1110/ps.8.3.587 PMC2144280

[B17] Milner-White E Ross BM Ismail R Belhadj-Mostefa K Poet R One type of gamma-turn, rather than the other gives rise to chain-reversal in proteins J Mol Biol 1988 204 3 777 782 10.1016/0022-2836(88)90368-3 3225851

[B18] Milner-White EJ Beta-bulges within loops as recurring features of protein structure Biochim Biophys Acta 1987 911 2 261 265 3801498 10.1016/0167-4838(87)90017-3

[B19] Overington J Donnelly D Johnson MS Sali A Blundell TL Environment-specific amino acid substitution tables: tertiary templates and prediction of protein folds Protein Sci 1992 1 2 216 226 1304904 10.1002/pro.5560010203 PMC2142193

[B20] Overington J Johnson MS Sali A Blundell TL Tertiary structural constraints on protein evolutionary diversity: templates, key residues and structure prediction Proc Biol Sci 1990 241 1301 132 145 10.1098/rspb.1990.0077 1978340

[B21] Worth CL Blundell TL Satisfaction of hydrogen-bonding potential influences the conservation of polar sidechains Proteins 2009 75 2 413 429 10.1002/prot.22248 18837037

[B22] Slingsby C Driessen HP Mahadevan D Bax B Blundell TL Evolutionary and functional relationships between the basic and acidic beta-crystallins Exp Eye Res 1988 46 3 375 403 10.1016/S0014-4835(88)80027-7 3350075

[B23] Eswar N Ramakrishnan C Deterministic features of side-chain main-chain hydrogen bonds in globular protein structures Protein Eng 2000 13 4 227 238 10.1093/protein/13.4.227 10810153

[B24] Vijayakumar M Qian H Zhou HX Hydrogen bonds between short polar side chains and peptide backbone: prevalence in proteins and effects on helix-forming propensities Proteins 1999 34 4 497 507 10.1002/(SICI)1097-0134(19990301)34:4<497::AID-PROT9>3.0.CO;2-G 10081962

[B25] Bordo D Argos P The role of side-chain hydrogen bonds in the formation and stabilization of secondary structure in soluble proteins J Mol Biol 1994 243 3 504 519 10.1006/jmbi.1994.1676 7966276

[B26] Mizuguchi K Deane CM Blundell TL Overington JP HOMSTRAD: a database of protein structure alignments for homologous families Protein Sci 1998 7 11 2469 2471 10.1002/pro.5560071126 9828015 PMC2143859

[B27] Harper ET Rose GD Helix stop signals in proteins and peptides: the capping box Biochemistry 1993 32 30 7605 7609 10.1021/bi00081a001 8347570

[B28] Serrano L Sancho J Hirshberg M Fersht AR Alpha-helix stability in proteins. I. Empirical correlations concerning substitution of side-chains at the N and C-caps and the replacement of alanine by glycine or serine at solvent-exposed surfaces J Mol Biol 1992 227 2 544 559 10.1016/0022-2836(92)90906-Z 1404368

[B29] Nicholson H Anderson DE Dao-pin S Matthews BW Analysis of the interaction between charged side chains and the alpha-helix dipole using designed thermostable mutants of phage T4 lysozyme Biochemistry 1991 30 41 9816 9828 10.1021/bi00105a002 1911773

[B30] Adzhubei AA Sternberg MJ Conservation of polyproline II helices in homologous proteins: implications for structure prediction by model building Protein Sci 1994 3 12 2395 2410 10.1002/pro.5560031223 7756993 PMC2142760

[B31] Hamill SJ Cota E Chothia C Clarke J Conservation of folding and stability within a protein family: the tyrosine corner as an evolutionary cul-de-sac J Mol Biol 2000 295 641 649 10.1006/jmbi.1999.3360 10623553

[B32] Mizuguchi K Deane CM Blundell TL Johnson MS Overington JP JOY: protein sequence-structure representation and analysis Bioinformatics 1998 14 7 617 623 10.1093/bioinformatics/14.7.617 9730927

[B33] Hutchinson EG Thornton JM PROMOTIF--a program to identify and analyze structural motifs in proteins Protein Sci 1996 5 2 212 220 8745398 10.1002/pro.5560050204 PMC2143354

[B34] Cubellis MV Cailliez F Lovell SC Secondary structure assignment that accurately reflects physical and evolutionary characteristics BMC Bioinformatics 2005 6 Suppl 4 S8 10.1186/1471-2105-6-S4-S8 16351757 PMC1866377

[B35] DeLano WL The PyMOL Molecular Graphics System 2002 Palo Alto, CA, USA: DeLano Scientific

