# Supplementary material for: On the evolutionary conservation of hydrogen bonds made by buried polar amino acids: the hidden joists, braces and trusses of protein architecture
Source: BMC Evol Biol. 2010 May 31;10:161. doi: 10.1186/1471-2148-10-161 (PMC2892493; doi:10.1186/1471-2148-10-161)
Supplement: Additional file 1 — Table of the 131 non-redundant families which were used in the analysis. [file 1471-2148-10-161-S1.DOC]

**Additional Table S1:** The 131 families having five or more members which were used in the analysis.

| **HOMSTRAD families** |
| --- |
| 6,7-dimethyl-8-ribityllumazine synthase |
| ABC transporter |
| Acetyltransferase (GNAT) family |
| alcohol dehydrogenase |
| Aldehyde oxidase and xanthine dehydrogenase, domains 1-2 |
| Aldehyde oxidase and xanthine dehydrogenase, domains 3-4 |
| aldo/keto reductase |
| alpha beta-hydrolase |
| amino acid dehydrogenase |
| aminotransferases class-I |
| annexin |
| Anticodon binding domain |
| Aspartate/ornithine carbamoyltransferase |
| aspartic proteinase |
| azurin/plastocyanin |
| beta-lactamase |
| beta-lactamase class B |
| beta/gamma crystallins |
| Biotin-requiring enzymes |
| C-type lectin |
| calcium-binding protein -- calmodulin-like |
| calcium-binding protein -- parvalbumin-like |
| Chalcone and stilbene synthases |
| Chitin binding domain |
| Class II histocompatibility antigen, C-terminal domain |
| Class II histocompatibility antigen, N-terminal domain |
| Cu/Zn superoxide dismutase |
| Cyclodextrin glycosyltransferase |
| cyclophilin (peptidylprolyl isomerase) |
| cytochrome b5 |
| cytochrome c |
| cytochrome p450 |
| cytochrome-c3 |
| DNA-binding homeodomain |
| epidermal growth factor-like domain |
| Eukaryotic-type carbonic anhydrase |
| fatty acid binding protein-like |
| Fe/Mn superoxide dismutase |
| ferredoxin (2Fe-2S) |
| ferredoxin (4Fe-4S) |
| fibronectin type III domain |
| flavodoxin |
| Gelsolin homology domain |
| globin |
| glutathione S-transferase |
| glyceraldehyde 3-phosphate dehydrogenase |
| glycosyl hydrolase family 1 |
| glycosyl hydrolase family 10 |
| glycosyl hydrolase family 11 |
| glycosyl hydrolase family 13 |
| glycosyl hydrolase family 22 (lysozyme) |
| glycosyl hydrolase family 5 |
| glycosyl hydrolases family 18 |
| GTP-binding protein |
| Haloperoxidase |
| Helicase conserved C-terminal domain |
| Helix-loop-helix DNA-binding domain |
| high potential iron-sulfur protein |
| Histidine kinase-, DNA gyrase B-, and HSP90-like ATPase |
| histocompatibility antigen-binding domain |
| HMG (high mobility group) box |
| hormone receptor (DNA-binding domain) |
| immunoglobulin domain -- C1 set - constant immunoglobulin |
| immunoglobulin domain -- C1 set - constant non immunoglobulin |
| immunoglobulin domain -- I set |
| immunoglobulin domain -- V set - immunoglobulin heavy chain |
| immunoglobulin domain -- V set - immunoglobulin light chain |
| immunoglobulin domain -- V set - variable non immunoglobulin |
| integrin I-domain |
| interleukin 1-beta-like growth factor |
| interleukin 8-like protein:11 |
| isocitrate and isopropylmalate dehydrogenase |
| kringle domain |
| lactate/malate dehydrogenase |
| legume lectin l |
| Ligand-binding domain of nuclear hormone receptors |
| lipocalin |
| long-chain scorpion toxin |
| Lyase |
| matrix metalloproteinase |
| Myb-like DNA-binding domain |
| NADH ubiquinone oxidoreductase, 20 Kd subunit |
| nitrogenase molybdenum-iron protein, alpha and beta chains |
| nucleotide kinase |
| pancreatic lipase |
| pancreatic ribonuclease |
| Papain family cysteine proteinase |
| PDZ Domain |
| peroxidase |
| PH domain |
| phospholipase A2 |
| phycocyanin |
| picornavirus coat proteins |
| plant lipid-transfer proteins |
| plant virus coat protein |
| profilin |
| Proteasome A-type and B-type |
| pyridine nucleotide-disulphide oxidoreductases class-I |
| reductases |
| Response regulator receiver domain |
| retroviral proteinase |
| Rhodanese-like domain |
| Ribosome inactivating protein |
| Ribulose bisphosphate carboxylase large chain |
| RNA recognition motif. (a.k.a. RRM, RBD, or RNP domain) |
| S-100/ICaBP type calcium binding protein |
| S-lectin |
| serine proteinase - bacterial |
| serine proteinase - eukaryotic |
| serine proteinase inhibitor -- Kunitz-type |
| serine proteinase inhibitor -- serpin-type |
| Serine/Threonine protein kinases, catalytic domain |
| short-chain dehydrogenases/reductases |
| snake toxin:20 |
| soybean trypsin inhibitor (Kunitz) |
| Src homology 2 domains |
| Src homology 3 domains |
| Staphylococcal/Streptococcal toxin |
| subtilase |
| Sulfotransferase protein |
| thymidylate synthase |
| TNF(Tumor Necrosis Factor) family |
| transferrin: 7 |
| Transforming growth factor-beta (TGF-beta) |
| triose phosphate isomerase |
| tRNA synthetases class II (G, H, P and S) |
| Tyrosine kinase, catalytic domain |
| ubiquitin conjugating enzyme |
| xylose isomerase |
| zinc finger -- CCHH-type |
| Zinc-binding domain present in Lin-11, Isl-1, Mec-3. |
