# Supplementary material for: On the evolutionary conservation of hydrogen bonds made by buried polar amino acids: the hidden joists, braces and trusses of protein architecture
Source: BMC Evol Biol. 2010 May 31;10:161. doi: 10.1186/1471-2148-10-161 (PMC2892493; doi:10.1186/1471-2148-10-161)
Supplement: Additional file 2 — Table of the families and their members that were used in the analysis. [file 1471-2148-10-161-S2.DOC]

**Additional Table S2:** The 65 families and their members which were identified as having entirely conserved and buried polar residues forming hydrogen bonds to mainchain atoms.

| **Family** | **Family Members** | **Ali Pos** |
| --- | --- | --- |
| Zinc-binding domain present in Lin-11, Isl-1, Mec-3. | 1ctl, 1qli, 1a7i, 1b8ta1, 1iml | 11, 38 |
| immunoglobulin domain -- C1 set - constant non immunoglobulin | 1hsaa, 3hlaa, 2vaba, 2vabb, 1b0gb | 87 |
| glycosyl hydrolase family 1 | 1cbg, 1e73m, 1hxja, 1ug6a, 1gnxa, 1e4ia, 1qoxa, 1qvba | 152 |
| Serine/Threonine protein kinases, catalytic domain | 1csn, 1ckia, 1a06, 1phk, 1cdka, 1koa, 1koba, 1tkia, 1jnk, 1p38, 3erk, 1hcl, 1blxa, 1lr4a, 1b6cb | 282 |
| alcohol dehydrogenase | 3huda, 1cdoa, 1teha, 2ohxa, 1d1ta | 37, 46, 49, 104, 112, 133, 144, 146, 171, 212, 268, 283 |
| serine proteinase inhibitor -- serpin-type | 2ach, 1qlpa, 1atha, 1atta, 1hle, 1ovaa, 1a7ca, 1sek | 161, 192 |
| high potential iron-sulfur protein | 1hpi, 2hipa, 1ckua, 1isua, 3hipa | 19, 43, 46, 80 |
| profilin | 1acf, 2acg, 1ypra, 1a0k, 1awia | 110 |
| S-lectin | 1hlca, 1slta, 1gana, 3gala, 1a3k | 66 |
| serine proteinase - bacterial | 2sfa, 2sga, 3sgbe, 1hpga, 2alp | 18, 34, 67, 149 |
| soybean trypsin inhibitor (Kunitz) | 1tie, 1wba, 1avwb, 1avac, 4wbca | 8, 20, 147 |
| Chalcone and stilbene synthases | 1ee0a, 1i88a, 1mzja, 1hzpa, 1ub7a, 1hnja | 68, 131, 217, 349 |
| NADH ubiquinone oxidoreductase, 20 Kd subunit | 1frfs, 2frva, 1e3da, 1h2rs, 1cc1s | 20, 76, 121, 194, 207, 232, 238, 243, 266, 269 |
| serine proteinase - eukaryotic | 1bbr, 1ppb, 1hcga, 1kigh, 1fxya, 2ptn, 1mcta, 1trma, 1a0ja, 2tbs, 1ab9, 1ton, 2pka, 1npma, 1azza, 3est, 1a0la, 1lmwb, 1a5ia, 1a5ha, 1fuja, 1hnee, 1a7s, 3rp2a, 1klt, 1dfpa, 1sgt | 248 |
| Aldehyde oxidase and xanthine dehydrogenase, domains 1-2 | 1hlra, 1dgja, 1jroa, 1fo4a, 1n62a, 1ffva | 49, 149, 152 |
| Aldehyde oxidase and xanthine dehydrogenase, domains 3-4 | 1dgja, 1hlra, 1fo4a, 1jrob, 1ffvb, 1n62b | 303, 338, 591, 732 |
| Rhodanese-like domain | 1e0ca1, 1rhs1, 1c25, 1qb0a, 1e0ca2, 1rhs2 | 52 |
| kringle domain | 2hppp, 2hpqp, 1tpka, 1kdu, 1pkr, 1pk4, 2pf1, 5hpga, 3kiv | 29, 60 |
| Aspartate/ornithine carbamoyltransferase | 3csua, 2otca, 1orta, 1a1s, 1otha | 129, 144, 147, 278 |
| cytochrome c | 1yea, 1ycc, 2pcbb, 5cytr, 1ccr, 1cry, 1hroa, 1cxc, 1c2ra, 155c, 2c2c | 27 |
| immunoglobulin domain -- V set - immunoglobulin light chain | 2fb4lv, 3bjllv, 2rhelv, 7fablv, 2mcglv, 8fablv, 3hfllv, 1baflv, 2fbjlv, 1reilv, 1igmlv, 1dfblv, 1fdllv, 1bvka, 1ar1lv, 6fablv, 1mamlv, 1jhllv, 4fablv, 1igflv, 1mcplv, 1hillv, 1bbdlv, 1lvelv, 1ncalv, 3hfmlv | 6, 92 |
| annexin | 1avha, 2ran, 1ala, 1aeia, 1axn, 1ann | 53 |
| isocitrate and isopropylmalate dehydrogenase | 1ipd, 1a05a, 2ayqa, 1cnza, 3icd | 164, 314, 371 |
| beta-lactamase | 4blma, 3blm, 1btl, 1mfo, 1bul, 1bsg, 1bza, 1shva | 110, 159, 160 |
| pancreatic ribonuclease | 7rsa, 1bsr, 1rra, 1b1ia, 1agi, 1bc4 | 82 |
| aspartic proteinase | 3app, 4ape, 2apr, 5pep, 1psn, 4cms, 1lyaa, 1bbs, 1smra, 2jxra, 1mpp, 2asi, 1am5 | 38, 112, 222, 249 |
| Sulfotransferase protein | 1cjma, 1aqua, 1efha, 1fmja, 1nsta | 247 |
| matrix metalloproteinase | 1mnc, 1hfc, 1mmpa, 1bqoa, 456ca, 1bqqm | 42, 107, 110, 112, 167 |
| beta/gamma crystallins | 1elpa, 1a45, 1a5da, 4gcr, 2bb2, 1prs, 1bd7a | 42, 88, 138, 183 |
| glycosyl hydrolase family 10 | 1exp, 1clxa, 1xyza, 1bg4, 1taxa | 139, 185, 193, 262, 300 |
| pancreatic lipase | 1etha, 1lpbb, 1hpla, 1rp1, 1bu8a | 23, 77, 102, 108, 122, 143, 153, 158, 173, 178, 194, 197, 205, 206, 227, 268, 275, 342, 405 |
| alpha beta-hydrolase | 1maha, 2ace, 1clea, 1trh, 1thg, 2bce | 117, 176, 177, 196, 201, 234 |
| glycosyl hydrolase family 11 | 1yna, 1xypa, 1xnb, 1xyn, 1bk1 | 131 |
| cytochrome-c3 | 2cdv, 2cym, 1wad, 3cyr, 2cy3, 1aqe | 40, 68, 100, 104 |
| legume lectin l | 2ltn, 1len, 1lgc, 1loe, 1lte, 2pela, 5cna, 1lec, 1sbf, 1wbla, 1avba, 1lu1 | 145, 234 |
| Lyase | 1jswa, 1fura, 1dcna, 1aosa, 1yfm | 148, 152 |
| subtilase | 1gt91, 1ga6a, 1dbia, 1thm, 1bh6a, 1csee, 1scja, 1lw6e, 1gci, 1ea7a, 1ic6a | 327 |
| interleukin 1-beta-like growth factor | 1afca, 2afga, 2fgf, 2mib, 1i1b, 1irax | 117 |
| glyceraldehyde 3-phosphate dehydrogenase | 1hdgo, 1gd1o, 1cero, 1gypa, 1ggao, 3gpdr, 1gpdg, 1gado | 162, 335, 336, 340 |
| Ribulose bisphosphate carboxylase large chain | 1gk8a, 8ruca, 1rbla, 1bxna, 1bwva, 1geha, 5ruba | 61, 144, 208, 246, 332, 335, 426 |
| xylose isomerase | 1dxia, 1xyaa, 6xia, 1xima, 4xiaa, 1bxba | 17, 141, 183, 215, 221, 247, 249 |
| integrin I-domain | 1lfaa, 1ido, 1atza, 1auq, 1aoxa | 23 |
| Ribosome inactivating protein | 1apa, 1qcia, 1abra, 1fmp, 1mrj, 1mrg, 1cf5a | 21, 225 |
| Papain family cysteine proteinase | 1mema, 1icfa1, 8pcha, 1gece, 1ppo, 1ppn, 1yal, 1cqda, 2act, 1aim, 1thea, 1huca, 1deua | 33 |
| PDZ Domain | 1be9a, 1pdr, 1qava, 1qaua, 1i16, 1kwaa | 63 |
| short-chain dehydrogenases/reductases | 1fmca, 1cyda, 1hdca, 1ybva, 1bdb, 1ae1a, 2ae1, 1enp, 1dfia, 1zid, 1fds, 1sep, 1dhr | 225 |
| hormone receptor (DNA-binding domain) | 2nlla, 1hra, 2nllb, 1hcp, 1lata | 8 |
| calcium-binding protein -- parvalbumin-like | 1rtp1, 1pvaa, 5cpv, 1pal, 5pal, 1omd, 1a75a | 51 |
| Haloperoxidase | 1b6g, 1cqwa, 1ehya, 1cr6a, 1a8s, 1a88a, 1a8q, 1brt, 1c4xa | 55, 85, 92 |
| Cu/Zn superoxide dismutase | 1cbja, 1mfma, 1xsoa, 1srda, 1eso, 2apsa, 1bzoa | 49, 54, 98, 139 |
| phospholipase A2 | 1bp2, 1p2p, 1poba, 1ae7, 1buna, 1vip, 1vpi, 1aokb, 1psj, 1vapa, 1pp2r, 1jiaa, 1a2aa, 1cl5a, 1clpa, 1goda, 1ppa, 1bbc | 27, 44 |
| Class II histocompatibility antigen, C-terminal domain | 1fnga, 1fv1a, 1iaka, 1hdmb, 1fngb, 1fv1b, 1iakb, 1hdma | 86 |
| Eukaryotic-type carbonic anhydrase | 1ca2, 2cab, 1koqa, 3znc, 1znca | 22, 38, 39, 72, 107, 116, 117, 128, 214, 226, 262, 264 |
| Cyclodextrin glycosyltransferase | 1pama, 1d3ca, 1cgt, 1ciu, 1cyg, 1qhpa | 18, 23, 24, 56, 85, 108, 143, 144, 174, 210, 221, 235, 338, 344, 364, 365, 372, 373, 376, 377, 387, 411, 414, 435, 436, 448, 462, 471, 503, 557, 586, 599, 684, 698 |
| azurin/plastocyanin | 2azaa, 1dz0a, 1joi, 1nwpa, 1jzga, 1rkra, 1cuoa, 1adwa, 1bqk, 1paz, 1pmy, 1iuz, 7pcy, 2plt, 9pcy, 1ag6, 1plc, 1byoa, 1plb, 1bawa, 1nin, 1m9wa, 1bxva, 2b3ia, 1kdj, 1qhqa, 2raca, 1id2a, 1gy1a | 167 |
| aldo/keto reductase | 1ah4, 1ads, 1frb, 2alr, 1afsa, 1a80, 1qrqa | 180 |
| peroxidase | 1qpaa, 1lgaa, 1mn2, 1arp, 2cyp | 95, 144 |
| Fe/Mn superoxide dismutase | 1ar5a, 1idsa, 3mdsa, 1n0ja, 3sdpa, 1isaa, 1mmma, 1sssa | 86, 178 |
| cytochrome p450 | 1n97a, 1jpza, 1dt6a, 1gwia, 1jipa, 1lfka, 1jfba, 1io7a, 1cpt, 1n40a, 1qmqa, 1e9xa | 362, 365, 448 |
| Tyrosine kinase, catalytic domain | 1vr2a, 1fgka, 3lck, 1qcfa, 2src, 1byga, 1ir3a | 217, 218, 228, 273, 281 |
| thymidylate synthase | 4tms, 3tms, 1tis, 2tsra, 1bkpa | 7, 74 |
| glutathione S-transferase | 1hna, 5gsta, 1gsua, 1gta, 2fhea, 1fhe, 2gsra, 1glpa, 17gsa, 1guha, 1guka, 1gula, 1gtua, 3gtub | 169 |
| pyridine nucleotide-disulphide oxidoreductases class-I | 2tpra, 1ndaa, 3grs, 1gera, 1ojt, 1ebda, 3lada, 1lpfa, 1lvl, 1npx, 1trb | 362 |
| picornavirus coat proteins | 1tme, 2mev, 1bbt, 1r1a, 4rhv, 2plv | 477, 545, 552, 757 |
| Staphylococcal/Streptococcal toxin | 3seb, 1ste, 1bxta, 1esfa, 1an8, 3tss | 88, 186 |

Alignment positions containing the entirely conserved and buried polar residues are indicated.

The family members are indicated by their PDB code. Where given, the fourth letter in the PDB code refers to the chain identifier.
