# Supplementary material for: On the evolutionary conservation of hydrogen bonds made by buried polar amino acids: the hidden joists, braces and trusses of protein architecture
Source: BMC Evol Biol. 2010 May 31;10:161. doi: 10.1186/1471-2148-10-161 (PMC2892493; doi:10.1186/1471-2148-10-161)
Supplement: Additional file 3 — Figures S1 to S6 show the propensity of polar amino acids to form hydrogen bonds to mainchain atoms in the various architectural contexts analysed. [file 1471-2148-10-161-S3.DOC]

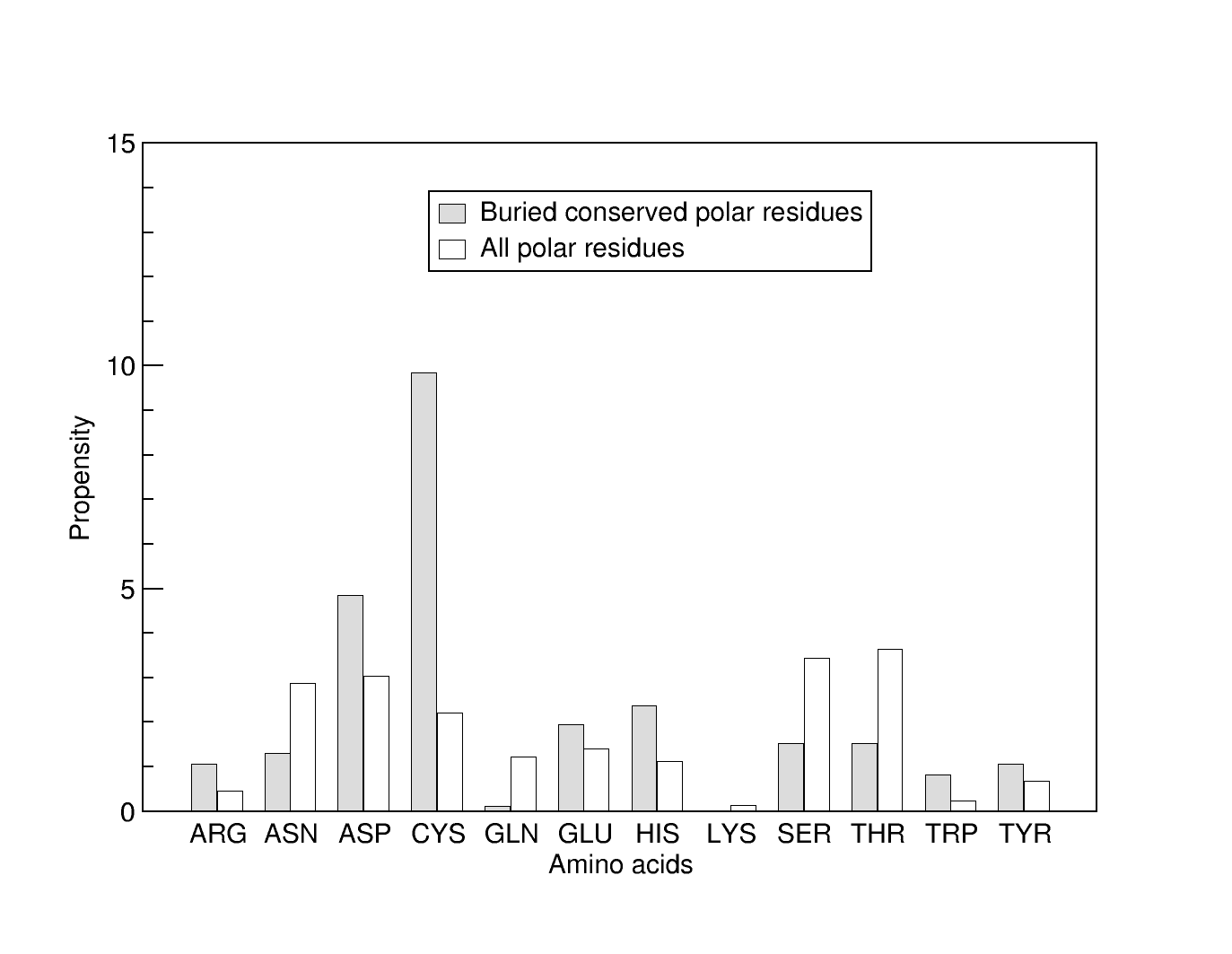


**A**


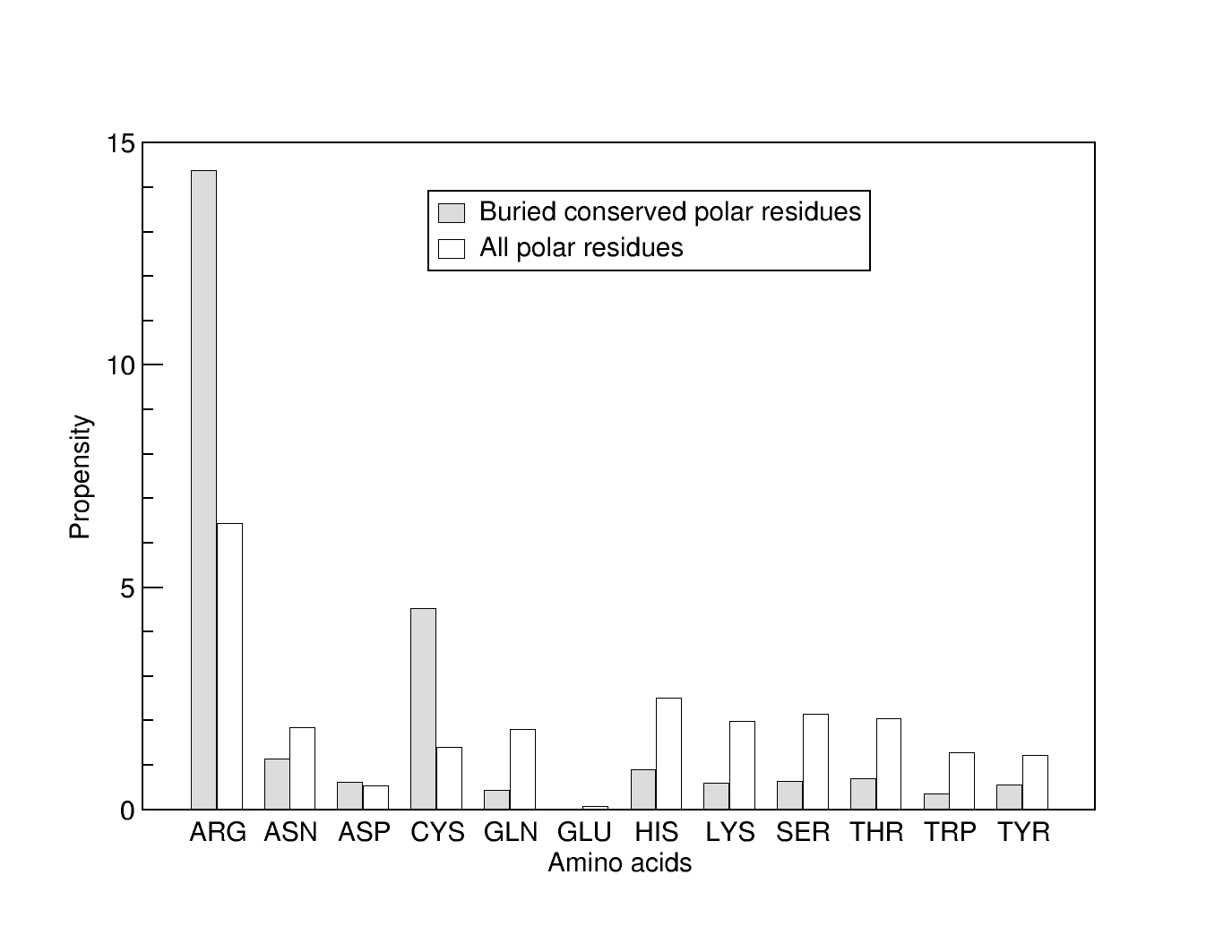


**B**

**Figure S1 - Propensity of polar residues to form sidechain hydrogen bonds to mainchain atoms within (A) helix N-termini and (B) helix C-termini.**

The propensities of conserved buried polar residues forming the above-mentioned hydrogen bonds are shown by grey bars. The propensities of all polar residues forming these interactions are shown by white bars.


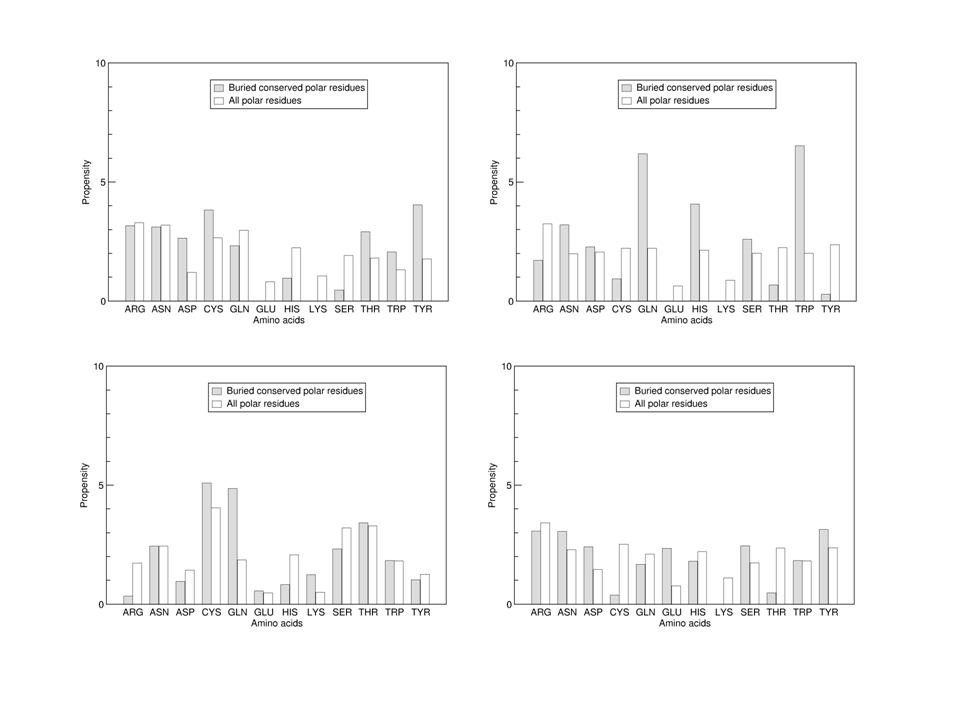


**Figure S2 - Propensity of polar residues to form sidechain hydrogen bonds to mainchain atoms A) within edge strands, B) from edge strands, C) within central strands and D) from central strands.**

**A**

**B**

**C**

**D**

The propensities of conserved buried polar residues forming the above-mentioned hydrogen bonds are shown by grey bars. The propensities of all polar residues forming these interactions are shown by white bars.


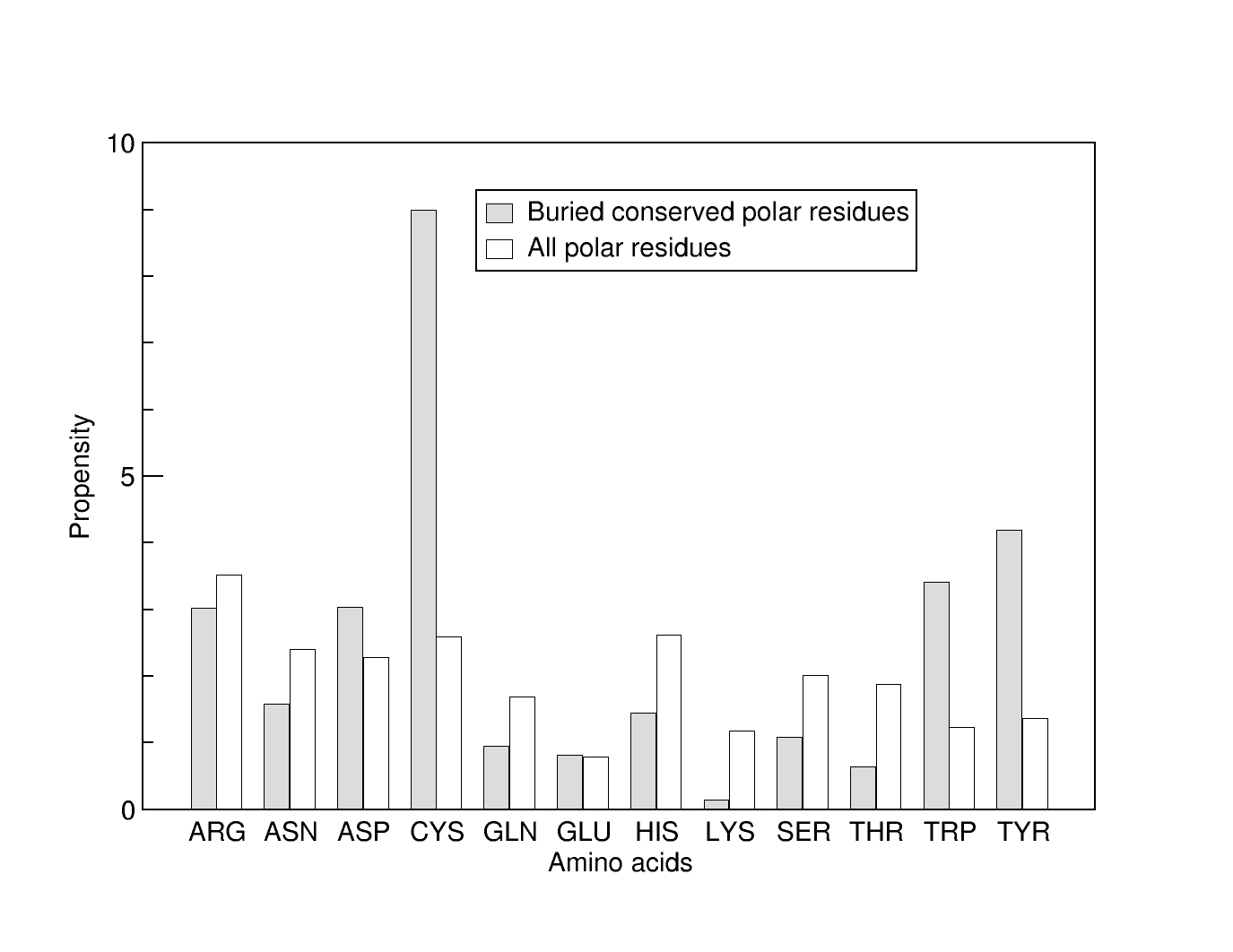


**Figure S3 - Propensity of polar residues to form sidechain hydrogen bonds to mainchain atoms within 3_10_ helices.**

The propensities of conserved buried polar residues forming hydrogen bonds to mainchain atoms within 3_10_ helices are shown by grey bars. The propensities of all polar residues forming hydrogen bonds to mainchain atoms within 3_10_ helices are shown by white bars.


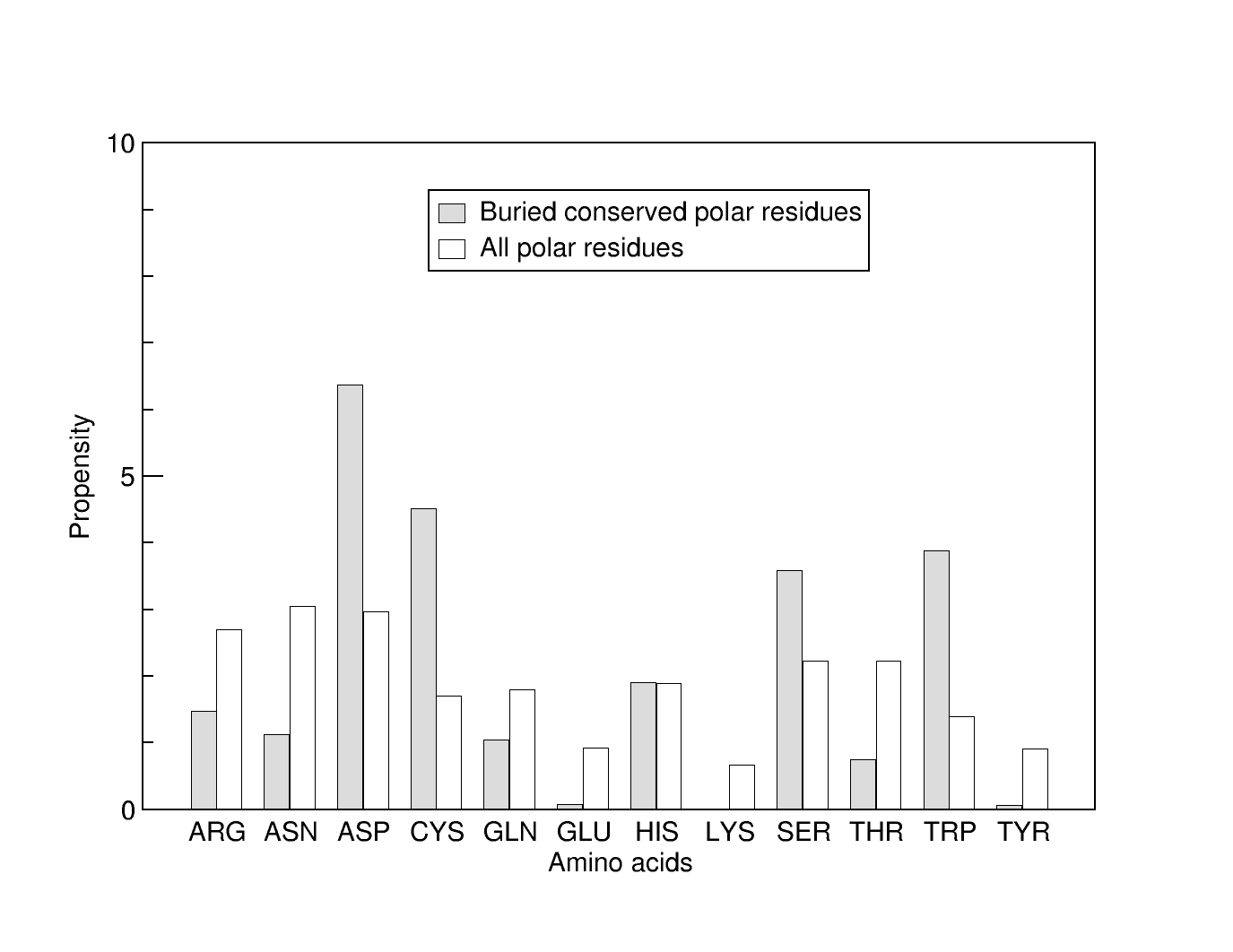


**Figure S4 - Propensity of polar residues forming sidechain hydrogen bonds to mainchain atoms within β-hairpins.**

The propensities of conserved buried polar residues forming sidechain hydrogen bonds to mainchain atoms within β-hairpins are shown by grey bars. The frequency of all polar residues forming hydrogen bonds to mainchain atoms within β-hairpins are shown by white bars.


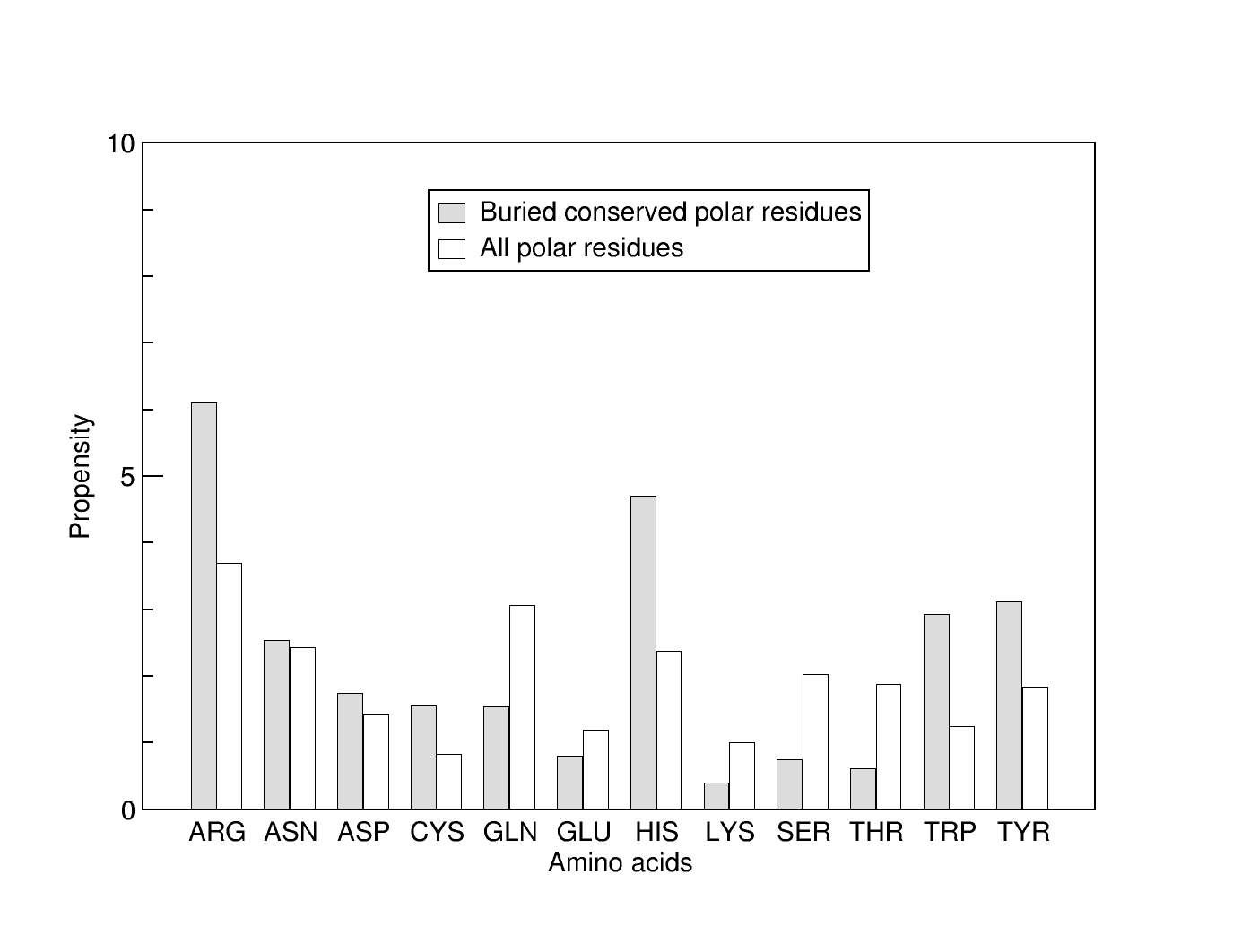


**Figure S5 - Propensity of polar residues forming sidechain hydrogen bonds to mainchain atoms within polyproline helices.**

The propensities of conserved buried polar residues forming the above-mentioned interactions are shown by grey bars. The frequency of all polar residues forming the above-mentioned interactions are shown by white bars.


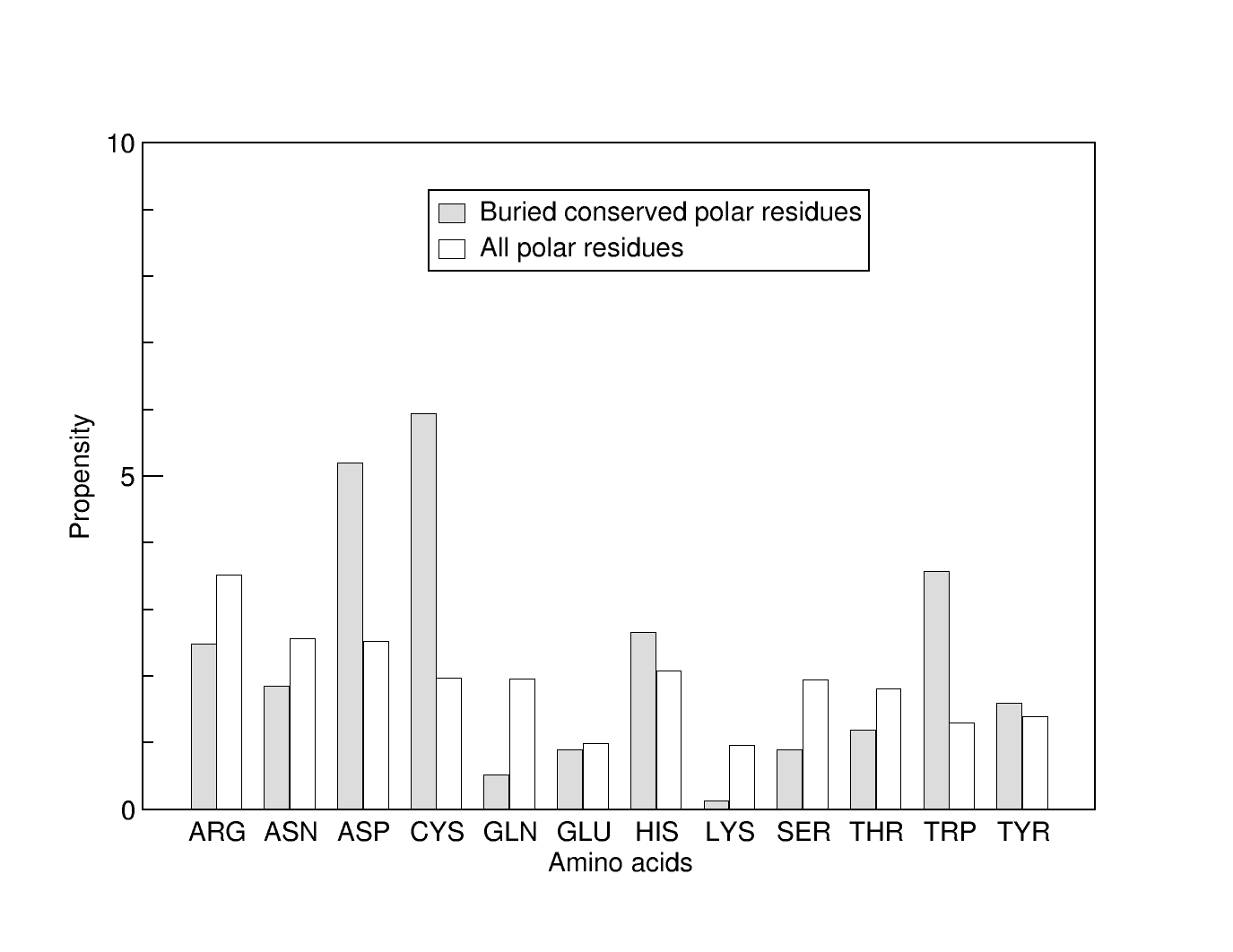


**Figure S6 - Propensity of polar residues forming sidechain hydrogen bonds to mainchain atoms within coils.**

The propensities of conserved buried polar residues forming sidechain hydrogen bonds to mainchain atoms within coils are shown by grey bars. The propensities of all polar residues forming these interactions are shown by white bars.
